# Capacity estimation and verification of quantum channels with arbitrarily correlated errors

**DOI:** 10.1038/s41467-017-00961-2

**Published:** 2018-01-02

**Authors:** Corsin Pfister, M. Adriaan Rol, Atul Mantri, Marco Tomamichel, Stephanie Wehner

**Affiliations:** 1 QuTech, Delft University of Technology, Lorentzweg 1, 2628 CJ Delft, The Netherlands; 2 Centre for Quantum Technologies, 3 Science Drive 2, Singapore, 117543 Singapore; 3 Kavli Institute of Nanoscience, Delft University of Technology, P.O. Box 5046, 2600 GA Delft, The Netherlands; 4 Singapore University of Technology and Design, 20 Dover Drive, Singapore, 138682 Singapore; 5 Centre for Quantum Software and Information, University of Technology Sydney, Broadway, NSW 2007 Australia

## Abstract

The central figure of merit for quantum memories and quantum communication devices is their capacity to store and transmit quantum information. Here, we present a protocol that estimates a lower bound on a channel’s quantum capacity, even when there are arbitrarily correlated errors. One application of these protocols is to test the performance of quantum repeaters for transmitting quantum information. Our protocol is easy to implement and comes in two versions. The first estimates the one-shot quantum capacity by preparing and measuring in two different bases, where all involved qubits are used as test qubits. The second verifies on-the-fly that a channel’s one-shot quantum capacity exceeds a minimal tolerated value while storing or communicating data. We discuss the performance using simple examples, such as the dephasing channel for which our method is asymptotically optimal. Finally, we apply our method to a superconducting qubit in experiment.

## Abstract (web summary)

Estimating the quantum capacity allows one to assess the performance of quantum memories, communication channels, repeaters as well as error correction schemes. Here, the authors show how to estimate and verify one-shot quantum capacity in the most general case of arbitrarily correlated errors.

## Introduction

One of the main obstacles on the way to quantum computers and quantum communication networks is the problem of noise due to imperfections in the devices. Noise is caused by uncontrolled interactions of the quantum information carriers with their environment. These interactions take place at all stages: when the carriers are processed, when they are transmitted, and when they are stored. Physicists and engineers spend large efforts in developing noise protection measures, and assessing their performance is crucial for the development of quantum information processing devices. In this article, we focus on the estimation of noise in the storage and transmission of the quantum information carriers, that is, we describe methods to assess quantum memory and quantum communication devices.

In the language of quantum information theory, memory and communication devices are described by a quantum channel, which is a function Λ that maps an input state *ρ*_in_ of the device to its output state *ρ*_out_=Λ(*ρ*_in_). In this unified description, assessing the noise in a quantum device reduces to estimating the decoherence of a quantum channel. One way to achieve this is through quantum process tomography^1^, which aims at completely determining the channel from measurement data (see e.g., refs. ^2,3^ for more recent works on tomography, and, e.g., refs. ^4,5^ for surveys on specific types of tomography). This comes with two major disadvantages. First, process tomography typically only works for channels that behave the same way in every run of the experiment (formalized by the i.i.d. assumption—for independent and identically distributed), or under some symmetry assumptions. This assumption is violated for many devices that are used in practice, which typically show correlated errors. Second, since process tomography aims at a complete characterization of the channel, it requires the collection of large amounts of data for many combinations of input states and measurement settings. A complete characterization of a channel is certainly useful (as all properties of the channel can be inferred from it), but it is very costly if the task at hand is to simply estimate a figure of merit of the channel. For quantum storage and quantum communication devices, a central figure of merit is the quantum capacity of the channel, which quantifies the amount of quantum information that can be stored or transmitted by the device^6^. While the deployment of a suitable error-correcting code requires knowledge of the specifics of the channel, an estimate of the quantum capacity is of great use when assessing the usefulness of the tested device.

In this work, we present a method to estimate the one-shot quantum capacity *Q* ^*ε*^(Λ) of a quantum channel Λ. While the quantum capacity *Q* only makes statements for devices that behave identically under many repeated uses, the one-shot quantum capacity *Q* ^*ε*^ applies to the more general case of devices with arbitrarily correlated errors. It quantifies the number of qubits that can be sent through the channel with a fidelity of at least 1 − *ε* in a single use of the device using the best possible error-correcting code (we will explain this in more detail in the next section). We present a protocol that allows to estimate *Q* ^*ε*^(Λ) from data obtained from simple measurements. In addition to dealing with arbitrarily correlated errors, it has the advantage of requiring fewer measurement settings than quantum process tomography.

Our method can also be used to assess whether a possibly imperfect error-correction scheme forms an improvement. This is the case if the error-corrected channel has a higher capacity than what we would otherwise expect. Similarly, our protocols can be employed to test whether a quantum repeater actually forms an improvement for sending quantum information, that is, whether it yields a higher quantum capacity than a direct quantum communication link.

## Results

### The one-shot quantum capacity

Noise can be modeled as a channel Λ, which is given as a map

1 $$\Lambda :{\cal S}\left( {\cal H} \right) \to {\cal S}\left( {\cal H} \right),$$

where ${\cal S}\left( {\cal H} \right)$ denotes the set of quantum states on the Hilbert space of the system that is being stored or transmitted. For reasons of illustration, we will discuss channels of storage devices here, but mathematically, nothing is different for communication devices. In the realm of communication, it is convenient to think of a sender (Alice) who wants to relay qubits to a receiver (Bob). For memory device, Alice and Bob simply label the input and output.

Consider a quantum memory device designed for storing a quantum system with Hilbert space ${\cal H}$ for some time interval Δ*t*. Ideally, it leaves the state of the system completely invariant over that time span, but real storage devices are always subject to noise. A measure for how well the channel Λ preserves the state of the system is obtained by minimizing the square of the fidelity between the input state $\left| \phi \right\rangle$ and the output state Λ(*ϕ*),

2 $$F\left( {\left| \phi \right\rangle ,\Lambda \left( \phi \right)} \right) = \sqrt {\left\langle \phi \right|\Lambda \left( \phi \right)\left| \phi \right\rangle } ,$$

over all possible input states $\left| \phi \right\rangle \in {\cal H}$,

3 $$\mathop {{\min }}\limits_{\left| \phi \right\rangle \in {\cal H}} {F^2}\left( {\left| \phi \right\rangle ,\Lambda (\phi )} \right) = \mathop {{\min }}\limits_{\left| \phi \right\rangle \in {\cal H}} \left\langle \phi \right|\Lambda \left( \phi \right)\left| \phi \right\rangle .$$

Low values of the quantity Eq. (3) imply that if the device is used without modification, then at least some states of the system are strongly affected by the channel, therefore introducing errors. However, this does not necessarily mean that the device is useless as a storage device, as this quantity does not account for the possibility that such errors can be corrected using quantum error correction (QEC).

**Fig. 1:**
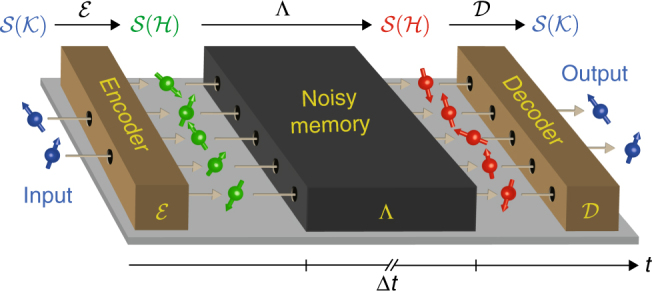
Time diagram of an error-corrected quantum memory. An error-correcting code can turn a noisy quantum memory for some system with a Hilbert space ${\cal H}$ into an approximately noise-free memory for some smaller system with a lower-dimensional Hilbert space ${\cal K}$. Such a code consists of an encoder ${\cal E}$, which is applied before the quantum memory, and a decoder ${\cal D}$, which is applied after the quantum memory. The encoder maps the state space ${\cal K}$ of the smaller system into a subspace ${\cal H}\prime \subseteq {\cal H}$ of the larger system that is stored by the quantum memory, so it implements an encoding channel ${\cal E}:{\cal S}\left( {\cal K} \right) \to {\cal S}\left( {\cal H} \right)$. The goal is to design the encoder such that the image ${\cal E}\left( {{\cal S}\left( {\cal K} \right)} \right) = {\cal S}\left( {{\cal H}\prime } \right) \subseteq {\cal S}\left( {\cal H} \right)$ is a subspace that is left approximately intact by the quantum memory, up to an operation that may have mapped it elsehwere. Then, the decoder can be chosen such that it implements a channel ${\cal D}:{\cal S}\left( {\cal H} \right) \to {\cal S}\left( {\cal K} \right)$ which maps that subspace back to the state space of the smaller system. This leads to an error-corrected memory for the smaller system which implements the channel ${\cal D} \circ \Lambda \circ {\cal E}:{\cal S}\left( {\cal K} \right) \to {\cal S}\left( {\cal K} \right)$. Note that this figure shows a time diagram, so the three devices are not necessarily placed in the same spatial order as they appear in the figure

An error-correcting code for a channel Λ consists of an encoding ${\cal E}$, which is applied before the channel, and a decoding ${\cal D}$, which is applied after the channel (see the explanations in Fig. 1). Together, these devices form an error-corrected quantum memory for a smaller system, implementing a channel

4 $${\cal D} \circ \Lambda \circ {\cal E}:{\cal S}\left( {\cal K} \right) \to {\cal S}\left( {\cal K} \right),$$

where ${\cal K}$ is the Hilbert space of the smaller system and where ο denotes the composition of maps. Instead of evaluating the quantity Eq. (3) for the channel Λ directly, it should be evaluated for such a corrected channel ${\cal D} \circ \Lambda \circ {\cal E}$. A figure of merit for the usefulness of the quantum memory is then given by the size of the largest system ${\cal K}$ that can be stored in the memory using such an error-correcting code. This is identical to the largest subspace ${\cal H}\prime \subseteq {\cal H}$ that is left approximately invariant by the memory, where the choice of encoding corresponds to the choice of subspace. This is quantified by the one-shot quantum capacity *Q* ^*ε*^(Λ), defined by^7,8^

5 $${Q^\varepsilon }\left( \Lambda \right)\,{\rm{:}} \!= {\rm{max}}\left\{ {{\rm{lo}}{{\rm{g}}_{\rm{2}}}\,m|{F_{{\rm{min}}}}\left( {\Lambda ,m} \right) \ge 1 - \varepsilon } \right\},$$

where

6 $${F_{{\rm{min}}}}\left( {\Lambda ,m} \right): = \mathop {{max}}\limits_{\scriptstyle{\cal H}\prime \subseteq {\cal H}\hfill\atop\\ \scriptstyle\dim ({\cal H}\prime ) = m\hfill} \mathop {{min}}\limits_{\left| \phi \right\rangle \in {\cal H}\prime } \left\langle \phi \right|\left( {{\cal D} \circ \Lambda } \right)\left( \phi \right)\left| \phi \right\rangle $$

and where the inner maximum is taken over all possible decoders ${\cal D}:{\cal S}\left( {\cal H} \right) \to {\cal S}\left( {\cal H} \right)$. This way, the one-shot quantum capacity corresponds to the maximal number of qubits that can be stored and retrieved with a fidelity of at least 1 − *ε* using the best possible error-correcting code.

The one-shot quantum capacity tells us strictly more than the asymptotic quantum capacity, in the sense that the latter can be obtained from the former:

7 $$Q\left( \Lambda \right) = \mathop {{\lim }}\limits_{\varepsilon \to 0} \mathop {{\lim }}\limits_{N \to \infty } \frac{1}{N}{Q^\varepsilon }\left( {{\Lambda ^{ \otimes N}}} \right).$$

The asymptotic quantum capacity is the number of qubits that can be transmitted or stored per use of a device with asymptotically vanishing error, in the limit where it is used infinitely often under the i.i.d. assumption. Therefore, it is an asymptotic rate, while the one-shot quantum capacity is the total number of qubits that can be transmitted or stored in a single use of a (possibly non-tensor product) channel, allowing some error $\varepsilon \ge 0$.

### One-shot quantum capacity estimation

Now that the one-shot quantum capacity is identified as the relevant figure of merit for quantum memory and communication devices, the question is whether we can estimate this quantity for a given device. We answer this question in the affirmative for the case where Λ is a channel that stores or communicates (arbitrarily many) qubits.

We present a simple protocol (see Protocol 1 in Table 1) that estimates a lower bound on the one-shot quantum capacity *Q* ^*ε*^(Λ) for an *N*-to-*N*-qubit channel Λ. Our protocol only requires the preparation and measurement of single qubit states in two bases. Specifically, even though it is known that the optimal encoder for a given channel Λ may require the creation of a highly entangled state, no entanglement is required to execute our test. For simplicity, we assume here that *N* is an even number (for more general cases, see Supplementary Notes 4–6). The protocol does not make any assumption on whether the qubits are processed sequentially, as in communication devices, or in parallel, as in storage devices (potentially with correlated errors in both cases). The data collection of the protocol is very simple. Alice and Bob agree on two qubit bases *X* and *Z*. These two bases should be chosen to be “incompatible”, in the sense that the preparation quality *q*, which is defined as

8 $$q = - {\rm{lo}}{{\rm{g}}_2}\mathop {{\max }}\limits_{i,j = 0,1} \left| {{{\left\langle {{i_X}\left| {{j_Z}} \right.} \right\rangle }}} \right|^2{\kern 1pt} ,$$

is as high as possible, where $\left| {{i_X}} \right\rangle$ and $\left| {{j_Z}} \right\rangle$ are eigenstates of *X* and *Z*, respectively. In the ideal case, where the two bases *X* and *Z* are mutually unbiased bases, such as the Pauli-*X* and *Z* basis, it holds that *q* = 1. Our protocol can be seen as exploiting the idea that the ability to transmit information in two complementary bases relates to a channel’s ability to convey (quantum) information^9,10^, which we show holds even with correlated noise. We remark that Pauli-*X* and *Z* basis have also been used to estimate the process fidelity of a quantum operation^11,12^ in the i.i.d. case, which however we are precisely trying to avoid here.

**Table 1:** Protocol 1: The estimation protocol

**One-shot quantum capacity estimation**
**Protocol parameter**
• $N \in {\Bbb N}$, even: total number of qubits
**The protocol**
• Alice chooses $s \in \left\{ {0,1} \right\}^N$ and $b \in \left\{ {X,Z} \right\}_{N/2}^N$ fully at random and communicates them to Bob, where
$$\{ X,Z \}_{N/2}^N = \{ b \in \{ X,Z \}^N | {\rm X},{\rm Z}, {\rm each}\,{\rm occur}\,N{/}2\,{\rm times}\,{\rm in}\,b \}.$$
• For each qubit slot *i* = 1, …., *N* of the channel, Alice prepares a test qubit *i* in the state *S*_*i*_ with respect to basis $b_i \in \left\{ {X,Z} \right\}$ and sends it through the channel to Bob.
• For each qubit *i* = 1, …., *N* that Bob receives, he measures test qubit *i* in the basis *b*_*i*_ and records the outcome $s_i^{\prime} \in \left\{ {0,1} \right\}$.
• Bob determine the error rates
$$e_x = \frac{2}{N}\mathop {\sum}\limits_{i \in I_X} s_i \oplus s{\prime}_i,\quad e_z = \frac{2}{N}\mathop {\sum}\limits_{i \in I_Z} s_i \oplus s{\prime}_i,$$
where
$$\begin{array}{l}I_X = \left\{ {i \in \left\{ {1, \ldots ,N} \right\}\left| {b_i = X} \right.} \right\},\\ I_Z = \left\{ {i \in \left\{ {1, \ldots ,N} \right\}\left| {b_i = Z} \right.} \right\}.\end{array}$$
• Knowing the two error rates *e*_*x*_ and *e*_*z*_, Bob determines a lower bound on the one-shot quantum capacity according to Theorem 1.

The bound for the capacity estimate is a function of the number of qubits *N*, the preparation quality *q*, the maximally allowed decoding error probability *ε* of *Q* ^*ε*^(Λ), the two measured error rates *e*_*x*_ and *e*_*z*_, and some probability *p* that quantifies the typicality of the protocol run (we will discuss this parameter in the Discussion section). More precisely, the bound is given as follows.

### Theorem 1

Let $N \in {{\Bbb N}_ + }$ be an even number, let *e*_*x*_ and *e*_*z*_ be error rates determined in a run of Protocol 1 where the used bases *X* and *Z* had a preparation quality of *q* (see Eq. (8) above). Then, for every *ε* > 0 and for every $p \in [0,1)$, it holds that

- either, the probability that at least one error rate exceeds *e*_*x*_ or *e*_*z*_, respectively, was higher than *p*,
- or the one-shot quantum capacity of the N-qubit channel Λ is bounded by

9 $$	 {Q^\varepsilon }\left( \Lambda \right) \ge \mathop {{\sup }}\limits_{\eta \in \left( {0,\sqrt {\varepsilon /2} } \right)} \\ 	 \left[ {N\left( {q - h\left( {{e_x} + \mu } \right) - h\left( {{e_z} + \mu } \right)} \right) - 2\,{\rm{lo}}{{\rm{g}}_2}\left( \kappa \right) - 4\,{\rm{lo}}{{\rm{g}}_2}\left( {\frac{1}{\eta }} \right) - 2} \right],$$

where *h* is the binary entropy function

10 $$h\left( x \right): = - x\,{\rm{lo}}{{\rm{g}}_2}\left( x \right) - \left( {1 - x} \right){\rm{lo}}{{\rm{g}}_2}\left( {1 - x} \right)$$

and *μ* and *κ* are given by

11 $$\mu = \sqrt {\frac{{N + 2}}{{{N^2}}}{\rm{ln}}\left( {\frac{{3 + \frac{5}{{\sqrt {1 - p} }}}}{{\sqrt {\varepsilon {\rm{/}}2} - \eta }}} \right)} ,\quad \kappa = 2{\left( {\frac{{3 + \frac{5}{{\sqrt {1 - p} }}}}{{\sqrt {\varepsilon {\rm{/}}2} - \eta }}} \right)^2}.$$

In the asymptotic limit where *N*→∞, the bound on the right hand side of inequality^9^ converges to $N\left( {q - h\left( {{e_x}} \right) - h\left( {{e_z}} \right)} \right)$. All the other terms can be seen as correction terms that account for finite-size effects. We will discuss this in more detail in the Discussion section below. One may wonder, why we do not also obtain an upper bound. First of all, there exist no way to distinguish noise in the rest of the experimental apparatus from the noise on the channel. Second and more significantly, however, fixing any estimation procedure, arbitrarily correlated noise can always conspire to defeat the procedure tricking us into believing the capacity is low, while actually it is quite high. An upper bound could be obtained under the assumption that the noise is i.i.d., but this is precisely what we wish to avoid here.

### One-shot capacity verification

Protocol 1 above estimates how much quantum information can be stored in a quantum memory device. This is of great use when the task is to figure out whether a device is potentially useful as a quantum memory device. When eventually, an error-correcting code is implemented, the corrected memory might be used without further testing.

In some cases, however, one wants to implement the memory with a means to verify its quality while using it. For example, one may suspect the quality of the memory to diminish (say, due to damage or overuse). In that case, the capacity estimation that was made before the implementation of the error-correcting code may no longer be valid. A method to verify that the quality of the memory is good enough for the implemented code may be required whenever it is used. Protocol 2, as given in Table 2, shows such a verification protocol.

**Table 2:** Protocol 2: The verification protocol

**One-shot quantum capacity verification**
**Protocol parameters**
• $N \in {\Bbb N}$: number of data qubits
• $e_x,e_z \in [0,1]$: tolerated error rate in *X*, *Z*
**The protocol**
• Alice chooses $s \in \left\{ {0,1} \right\}^{3N}$ and $b \in \left\{ {X,Z,D} \right\}_N^{3N}$ fully at random and communicates them to Bob, where
$$\left\{ {X,Z,D} \right\}_N^{3N} = \{ {b \in \left\{ {X,Z,D} \right\}^{3N} \left| {X,Z,D\,{\rm{occur}}\,N\,{\rm{times}}\,{\rm{in}}\,b} \right.} \}.$$
• For each qubit slot *i* = 1, …, 3*N* of the channel, if $b_i \in \left\{ {X,Z} \right\}$, Alice prepares a test qubit *I* in the state *s*_*i*_ with respect to basis $b_i \in \left\{ {X,Z} \right\}$ and sends it through the channel to Bob. If *b*_*i*_ = *D*, Alice uses the slot for a data qubit.
• For each qubit *i*=1, …, 3*N* that Bob receives, if $b_i \in \left\{ {X,Z} \right\}$, Bob measures test qubit *I* in the basis *b*_*i*_ and records the outcome $s{\prime}_i \in \left\{ {0,1} \right\}$. If *b*_*i*_ = *D*, Bob leaves the data qubit untouched.
• They determine the error rates
$$\gamma = \frac{1}{N}\mathop {\sum}\limits_{i \in I_X} s_i \oplus s_i^\prime ,\quad \lambda = \frac{1}{N}\mathop {\sum}\limits_{i \in I_Z} s_i \oplus s_i^\prime ,$$
where
$$\begin{array}{l}I_X = \left\{ {i \in \left\{ {1, \ldots ,3N} \right\}\left| {b_i = X} \right.} \right\},\\ I_Z = \left\{ {i \in \left\{ {1, \ldots ,3N} \right\}\left| {b_i = Z} \right.} \right\}.\end{array}$$
If $\gamma \le e_x$ and $\lambda \le e_z$, they continue with the conclusion below. Otherwise, they abort the protocol.
• They conclude that the one-shot quantum capacity of the channel Λ on the *N* data qubits is bounded as in Theorem 2.

The protocol assumes that Alice holds *N* data qubits that she wants to send to Bob in a way that allows her to verify the quality of the transmission. To this end, she uses a channel for 3*N* qubits and places her *N* data qubits in random slots of this channel. The other 2*N* slots are used for test qubits, half of which are prepared and measured in the *X* basis and half of which are prepared and measured in the *Z* basis (just as in the estimation protocol), while Alice and Bob leave the data qubits untouched. The error rates on the test bits allows to infer a bound on the capacity of the channel on the data qubits.

For this protocol, we denote the measured error rate in *X* by γ and the measured error rate in *Z* by *λ*. Bob checks whether these error rates exceed some tolerated values *e*_*x*_ and *e*_*z*_, respectively, which has been specified before the protocol run. If one or both error rates exceed the tolerated value, the protocol aborts because the transmission quality is considered too low. If both error rates are below their tolerated value, Bob concludes that the transmission was of high quality, in the sense that the channel on the data qubits had a high one-shot quantum capacity. This is stated more precisely in the following theorem.

### Theorem 2

Let $N \in {{\Bbb N}_ + }$, let *e*_*x*_, ${e_z} \in [0,1]$. Assume that Protocol 2 is run successfully without abortion, where the used bases *X* and *Z* had a preparation quality of *q*. Then, for every *ε* > 0 and for every $p \in [0,1)$, it holds that

- either, the probability that the protocol aborts was higher than *p*,
- or the one-shot quantum capacity of the channel Λ on the N data qubits is bounded by inequality Eq. (9), where *κ* is as in Eq. (11) and where *μ* is given by

12 $$\mu = \sqrt {\frac{{2\left( {N + 1} \right)}}{{{N^2}}}{\rm{ln}}\left( {\frac{{3 + \frac{5}{{\sqrt {1 - p} }}}}{{\sqrt {\varepsilon {\rm{/}}2} - \eta }}} \right)} {\kern 1pt} .$$

**Fig. 2:**
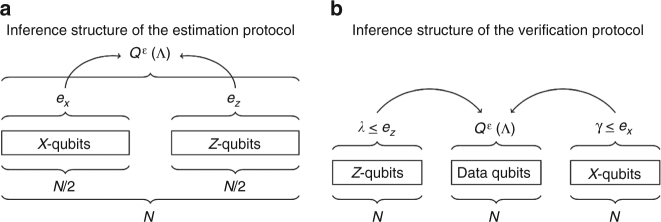
Comparison of the inference structures of the two protocols. **a** In the estimation protocol, all qubits are test qubits, and the goal is to estimate the capacity for the channel on all qubits. **b** In the verification protocol, one third of the qubits are data qubits that are left untouched. The remaining 2*N* qubits are test qubits, whose error rates allow to bound the capacity of the channel on the *N* data qubits

The bound for the verification protocol looks formally almost identical to the one for the estimation protocol, but there are three differences. First, the function *μ* has a different dependence on *N*, which is a consequence of the different structure of the protocol as explained in Fig. 2. Second, the error rates *e*_*x*_ and *e*_*z*_ are preset accepted error rates instead of calculated error rates from data, and the bound holds when the measured rates are below those preset values. Third, the probability *p* in the bound is the abort probability of the protocol. Hence, another way to read the statement of the theorem is that either the protocol succeeds (does not abort) with a probability at most 1 − *p*, or the capacity is indeed high. This again quantifies what we consider to be typical data: even if the channel is competely noisy and useless, there might be a tiny probability 1 − *p* that the observed error rates are nevertheless small. In this case, we saw highly atypical data. We will say more about this probability in the Discussion section. Recall, that in the verification protocol we use 3*N* rounds, hence there is no factor of 1/3 on *N* (see also Fig. 2).

### Experiment

We demonstrate the use of this protocol by implementing it on a Transmon qubit. The experiment is performed on qubit *A*_*T*_ previously reported in ref. ^13^. We measure a relaxation time of *T*_1_ = 18.5 ± 0.6 μs and a Ramsey dephasing time of $T_2^ \star = 3.8 \pm 0.3$ μs before performing the experiment. Readout of the qubit state is performed by probing the readout resonator with a microwave tone. The resulting transients are amplified using a traveling-wave parametric amplifier (TWPA)^14^ at the front end of the amplification chain. This results in a readout fidelity *F*_RO_ = 11 − (*p*_01 _+ *p*_10_)/2 = 98.0%, where *p*_01_ (*p*_10_) is the probability of declaring state 1 (0) when the input state was $\left| 0 \right\rangle$ $\left( {\left| 1 \right\rangle } \right)$ respectively. The qubit state is controlled using resonant microwave pulses.

**Fig. 3:**
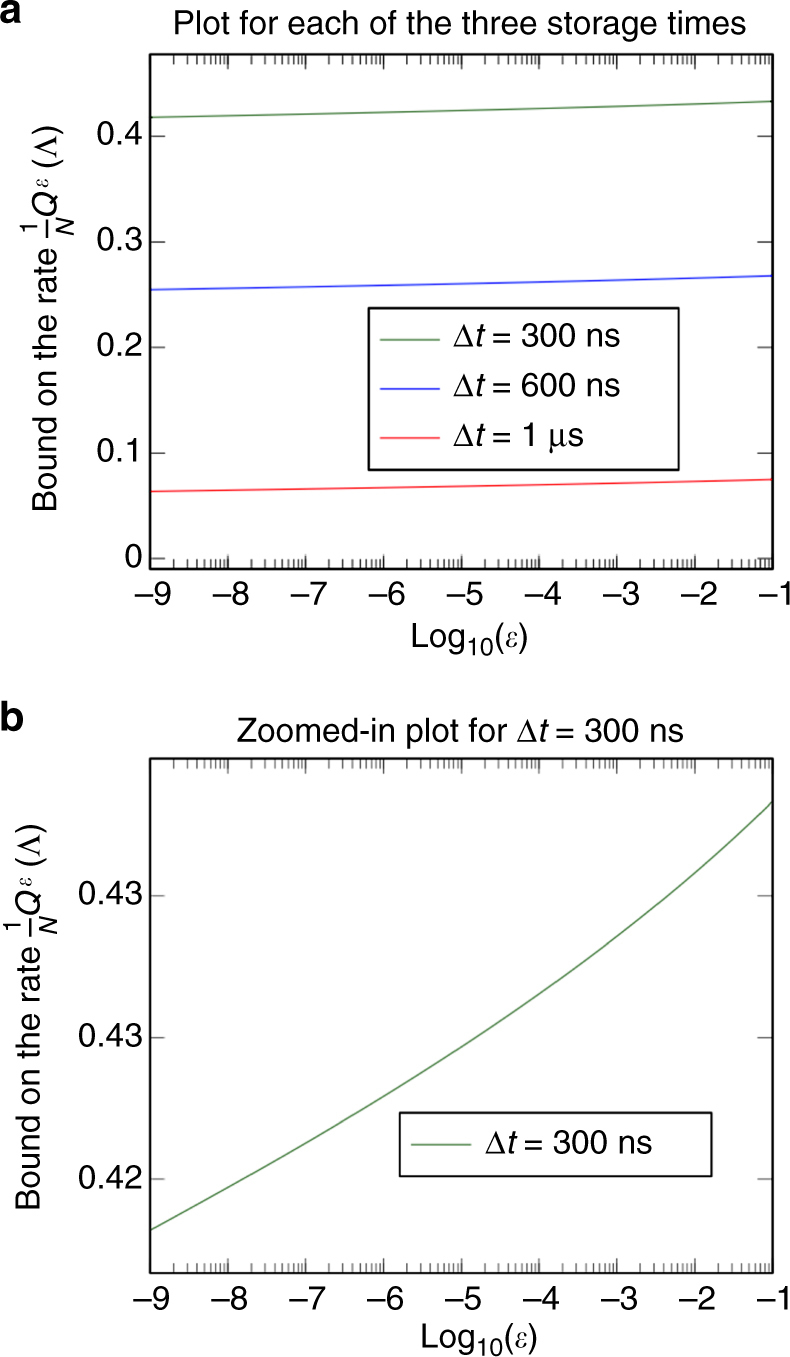
Bound on the rate for the experimental data as a function of *ε*. This figure shows the bound on the one-shot quantum capacity rate for the data gained in the transmon qubit. We pick *p* = 1/2, and use *q* = 0.9 as preparation quality to account for the experimental imperfections (see Supplementary Note 7 for details). **a** The experiment was carried out three times with different storage times Δ*t*, for each of which we plotted the bound resulting from the estimation protocol as a function of the decoding error probability *ε*. Since the number of qubit preparations and measurements was high (*N* = 1.04 × 10^6^), the dependence on *ε* is rather small. **b** For a better visibility of the ε-dependence, we show the plot for the shortest storage time separately and more zoomed-in in the direction of the bound

**Fig. 4:**
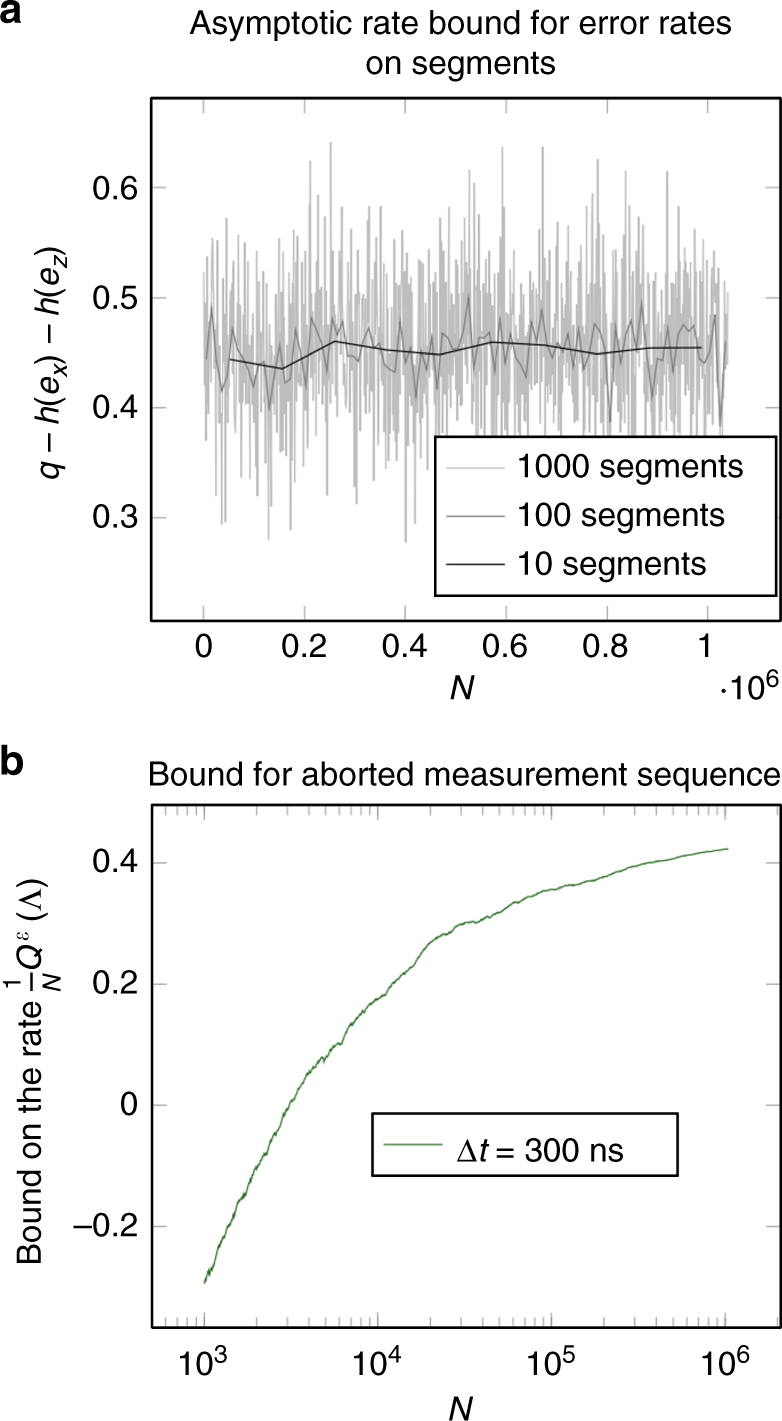
Error fluctuations across the measurements. Here we visualize the statistical fluctuations in the measurement outcomes over the course of the transmon qubit experiment. **a** For the experiment with Δ*t* = 300 ns, we split up the *N*=1.04 × 10^6^ sequential measurement outcomes into equally large and chronologically ordered segments and calculate the error rates *e*_*x*_ and *e*_*z*_ on each segment. For a meaningful and comparable quantity for comparison, we calculate the asymptotic bound $q - h\left( {{e_x}} \right) - h\left( {{e_z}} \right)$ for each of segment with *q* = 0.9, that is, the bound on the capacity rate that would be obtained if infinitely many measurements with the error rates as on the respective segments would be measured. As expected, the fluctuations decrease with the number of segments, or in other words, the larger the segments, the smaller the differences between them. Note that in contrast to all other plots, this is a linear plot. **b** For a glimpse on the cumulative effect of the fluctuations, we set 1000 logarithmically distributed “break points” and calculate the bound as if the experiment ended at each of those points where *q* = 0.9, *ε* = 10^−6^, and we pick *p* = 1/2. The resulting plot is to be compared with the plots in Fig. 5. The fluctuations that make the curve deviate from a smooth curve come from the fact that the measured error rates are not constant throughout the experiment, indicating that the noise affecting the transmon qubit is indeed unlikely to correspond to an i.i.d. process

The experiment implements Protocol 01 to estimate the capacity of the idling operation *I*(Δ*t*). We do this by generating 8000 pairs of random numbers corresponding to the bases $b \in \left\{ {X,Z} \right\}$ and states $s \in \left\{ {0,1} \right\}$. These are then used to generate pulse sequences that rotate $\left| 0 \right\rangle$ to the required state, and wait for a time Δ*t* before measuring the qubit in the *Z* basis and declaring a state. If the required state was in the *X* basis, a recovery pulse is applied that rotates the state to the *Z* basis before it is read out. This protocol is repeated 130 times, with a distinct randomization for each repetition, yielding a total of *N* = 1.04 × 10^6^ measurement outcomes in approximately one and a half hours. Results are reported in Fig. 3, which illustrates the estimate using the totality of the *N* outcomes for different values of *ε*. In Fig. 4 we furthermore plot variations in the error rate over time, as well as a bound for partial measurement sequences which highlight the (likely) non i.i.d. nature of the actual noise process affecting the qubits. We estimate *q* = 0.985 ± 0.047 (see Supplementary Note 7) before taking the data, but use *q* = 0.9 as a conservative estimate to account for a potential drift during the experimental run.

## Discussion

**Fig. 5:**
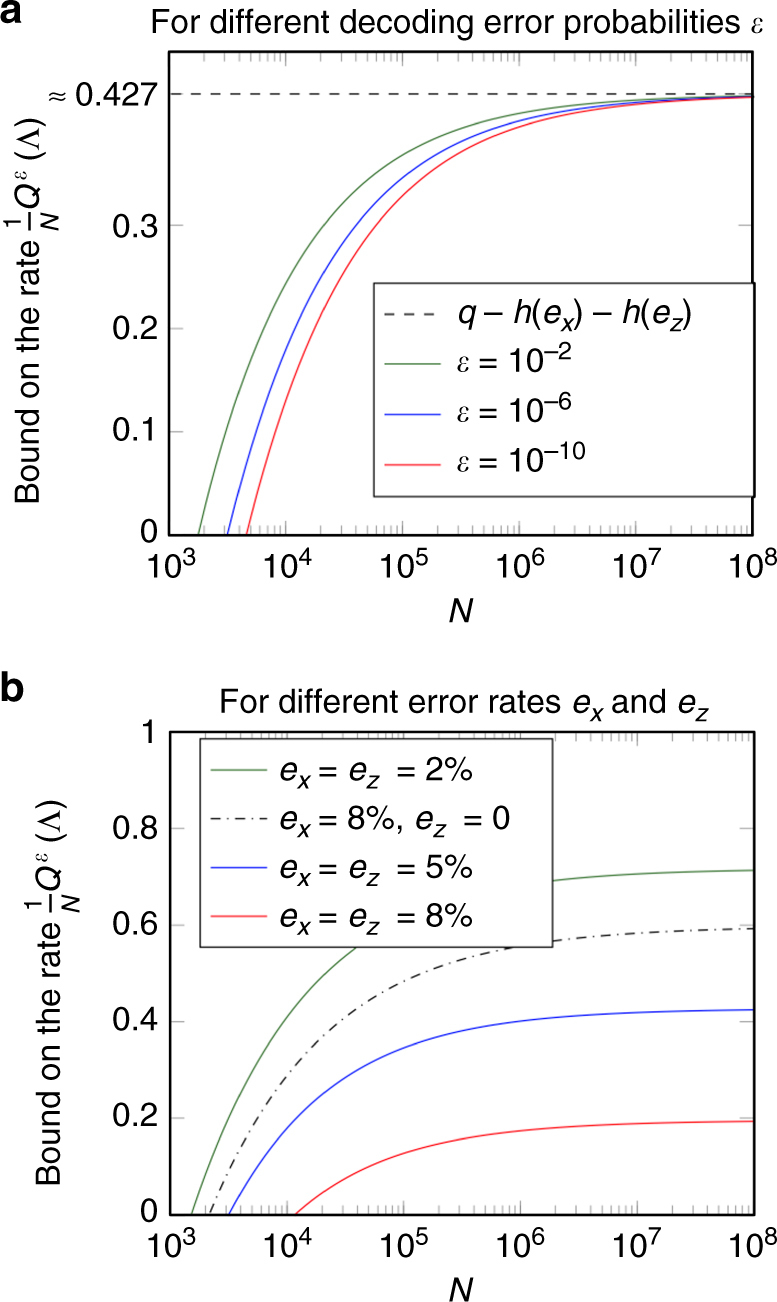
Bound on the rate for the capacity estimation protocol as a function of the number of qubits. This figure shows the bound on the one-shot quantum capacity for the estimation protocol expressed as a rate, that is, the right hand side of inequality^9^ divided by the number of qubits *N*. The plots show the bound as a function of *N* with the parameters as *q* = 1, and *p* = 1/2. **a** We plotted the bound for fixed error rates *e*_*x*_ = *e*_*z*_ = 5% for a few different values of *ε* in order to visualize the dependence on the decoding error probability. The lower the allowed decoding error probability *ε* is set, the higher the number of qubits needs to be in order to get a positive bound on the rate (note that the *N*-axis is logarithmic). In the asymptotic limit *N→*∞, the bound converges to $q - h\left( {{e_x}} \right) - h\left( {{e_z}} \right)$. If *q* = 1, this coincides exactly with the (asymptotic) capacity for some important classes of channels, such as depolarizing channels. This shows that our bound is asymptotically optimal, and therefore, improvements are only possible in the finite-size correction terms. **b** To see the dependence on the error rates, we plotted our bound for a fixed value of *ε* = 10^−6^ for a few different values of *e*_*x*_ and *e*_*z*_. The higher the error rate, the higher the number of qubits needs to be in order to achieve a positive rate. For every pair of error rates *e*_*x*_ and *e*_*z*_, the bound is monotonically increasing in *N* and converges to $q - h\left( {{e_x}} \right) - h\left( {{e_z}} \right)$. Therefore, the bound can only be positive when $q - h\left( {{e_x}} \right) - h\left( {{e_z}} \right)$ is positive, which yields an easy criterion for the potential usefulness of a channel with known error rates (although the full version of the bound with the correction terms is not hard to evaluate either)

In this section, we shall discuss our bound as a bound on the rate $\frac{1}{N}{Q^\varepsilon }\left( \Lambda \right)$, which quantifies the amount of quantum information that can be sent per qubit. This has the advantage that it makes comparisons easier. To discuss our bound on the capacity rate, we have plotted its value as a function of *N* in Fig. 5. We plotted the bound for the estimation protocol, but qualitatively, the bound for the verification protocol behaves identically, so our discussion applies to both protocols.

### Example dephasing channel

In order to assess the strength of our bound, it is helpful to consider some example channels. A particularly insightful example is the case where the channel Λ is given by *N* independent copies of a dephasing channel of strength $\alpha \in \left[ {0,1} \right]$, that is,

13 $$\Lambda = \Lambda _D^{ \otimes N},\quad {\Lambda _D}\left( \rho \right):\rho \; \mapsto \,\left( {1 - \frac{\alpha }{2}} \right)\rho + \frac{\alpha }{2}\sigma \,\rho \sigma ,$$

where *σ* denotes one of the qubit Pauli operators with respect to some basis. Of particular interest is the case where the dephasing happens with respect to one of the two bases *X* or *Z* in which Alice and Bob prepare and measure the test qubits. Let us assume that *σ* = *σ*_*Z*_. In order to see what happens when our estimation protocol is used in this case, we could simulate a protocol run and see what bound on the one-shot quantum capacity would be obtained. However, the estimation protocol does essentially nothing but determine the two error rates *e*_*x*_ and *e*_*z*_. The expected values of these rates can be readily obtained from Eq. (13). The error rate *e*_*z*_ vanishes, because dephasing in the *Z* basis leaves the *Z*-diagonal invariant. In the *X* basis the bits are left invariant with probability 1 − *α*/2, and flipped with probability *α*/2, so asymptotically *e*_*x*_ = *α*/2. Hence, for the dephasing channel, the estimation protocol is expected to yield the bound in inequality Eq. (9) with *e*_*z*_ = 0 and *e*_*x*_ = *α*/2.

### Asymptotic tightness of the bound

As one can see in Fig. 5, the bound on the one-shot quantum capacity, expressed as a rate, converges to $q - h\left( {{e_x}} \right) - h\left( {{e_z}} \right)$, which in the case of the dephasing channel is given by *q* − *h*(*α*/2). If we additionally assume that the bases *X* and *Z* are mutually unbiased (as are Pauli-*X* and *Z*), this is equal to 1 − *h*(*α*/2). This is precisely the (asymptotic) quantum capacity of the dephasing channel. This means that our bound on the one-shot quantum capacity is asymptotically tight; if our bound can be improved, then only in the finite-size correction terms. In particular, our bound cannot be improved by a constant factor. Since most estimates that enter the derivation of the bound are of the same type as the estimates used in modern security proofs of quantum key distribution (QKD)^15^, any possible improvements of the QKD security bounds would also lead to an improvement of our bound on the one-shot quantum capacity (if there is any). In this sense, our bound is essentially as tight as the corresponding security bounds for QKD in the finite regime.

### Measurement calibration

Above, we have assumed that Alice and Bob were very lucky: they set up their bases *X* and *Z* such that one of them is exactly aligned with the dephasing basis, and therefore optimally exploited the asymmetry of the channel. In general, since they do not know the channel whose capacity they estimate, they do not know about the direction of the asymmetry. Instead, they have to calibrate their devices by trying out several pairs of bases until they find one with low error rates. Otherwise, the bound on the one-shot quantum capacity that they infer is suboptimal. It is an interesting open question how such a calibration can be optimized.

### Example fully depolarizing channel

Another insightful example is the case where Λ is given by the channel which outputs the fully mixed state of *N* qubits, independently of the input state. The capacity of this channel is zero, yet with probability 2^−*N*^, Alice and Bob measure error rates *e*_*x*_ = *e*_*z*_ = 0. One may think that these vanishing error rates lead to a highly positive bound on the capacity, but this is not the case. As one can read in Theorem 1 and Theorem 2, the bound depends on a probability *p*, and the term 1−*p* corresponds precisely to the probability of such an unlikely case. In fact, for 1 − *p* = 2^−*N*^, the bound is never positive. This example shows that in the one-shot regime, a meaningful capacity estimation can only be made under the assumption that the observed data is not extremely atypical for the channel. However, this is only a problem for very low values of *N*: thanks to the natural logarithm in *μ* (see Eq. (11) above), the concern reduces to atypical events with an exponentially (in *N*) small probability. For reasonable numbers of *N*, the influence of *p* on the bound is negligible, except for extremely low values of 1 − *p*.

### Quantifying typicality

We remark that *p* is a parameter to choose before executing the estimation protocol, which essentially just defines what we consider atypical. The statement of the estimation theorem can then be understood as simply stating that either the observed data is highly atypical (as defined by the choice of *p*), or the capacity is indeed high. From a practical point of view, note that for any constant *p*, the bound is essentially independent of *p* for even relatively small values of *N* and *μ*→0 in (Eq. (11)) as *N*→∞. For this reason, we simply choose *p* = 1/2 in our plots as an illustration. Similarly, the maximally tolerated error rates *e*_*x*_ and *e*_*z*_ can be chosen freely when conducting the verification protocol. It is merely that the conclusions of the test depend on it, since we choose to abort—that is, draw no conclusion—if the observered error rates are higher than *e*_*x*_ and *e*_*z*_. The probability *p*_pass_ that we do not abort then corresponds to *p*_pass_ = 1 − *p*. So *p* also arises here, and corresponds to the probability of aborting, namely to the probability that data is produced which we do not regard as typical. Aborting may still seem like a different approach to the one taken during the verification protocol where we always draw a conclusion, but we can see that it is in fact exactly analogous: In the estimation protocol, Alice and Bob essentially decide to make a test in which *e*_*x*_ and *e*_*z*_ correspond exactly to the measured error rates instead of setting a maximum error rate ahead of time. Clearly, they will never abort in this case. Nevertheless, one can consider the probability that in any run, the measured error rates would stay below the rates that have been measured in this particular run of the test. This probability can be seen as a measure of typicality of the protocol, and corresponds precisely to *p*_pass_ if we were to execute the test again, but now fixing the error rates to what we observed. Hence, again *p* = 1 − *p*_pass_ which corresponds precisely to a quantification of such typicality.

For more information on the probability *p* and *e*_*x*_ and *e*_*z*_, see Supplementary Notes 5 and 6. Note that the need to characterize typicality of the data observed is not only given in our context of capacity estimation, but arises in all statistical tests on a finite sample, including quantum key distribution where security statements are formulated in an analogous fashion.

### Usage to assess quantum repeaters

An important challenge in the experimental realization of quantum repeaters is to demonstrate that they improve our ability to communicate compared to a direct fiber connection without a repeater. To demonstrate that they improve our ability to produce key, one proceeds by calculating the private capacity *P* of the direct communication channel without a repeater, followed by the implementation of a QKD protocol using the quantum repeater. If the rate *R* of generating key in the QKD protocol with the repeater satisfies *R* > *P*, then the quantum repeater improved our ability to produce encryption keys (see e.g., refs. ^16–18^). It turns out that it is in general harder to send qubits, then it is to produce key^19^. That is, the quantum capacity *Q* satisfies $Q \le P$, where the inequality is in general strict. Demonstrating that a repeater improves our ability to produce key thus does not allow us to draw conclusions on whether the repeater improves our ability for sending qubits without further analysis.

Our result now precisely allows one to perform such a demonstration, even in the regime of arbitrarily correlated noise while in fact being no more difficult than performing BB84 QKD^20^. Crucially, this means that in order to demonstrate a quantum repeaters ability to send qubits it is thus not necessary to perform quantum error correction (QEC) or entanglement distillation. First, one needs to calculate the quantum capacity $Q_{DF}^\varepsilon \left( N \right)$ of the direct fiber (DF) connection, or a bound $Q_{DF}^\varepsilon \left( N \right) \le B_{DF}^\varepsilon \left( N \right)$. We note that theoretical bounds $B_{DF}^\varepsilon \left( N \right)$ on the one-shot capacity $Q_{DF}^\varepsilon \left( N \right)$ are known for finite number of channel uses *N* and error ε, which are much tighter than employing the asymptotic capacity for *N*→∞ and *ε*→0^8,21^. We can now run our capacity estimation protocol over the quantum repeater link which yields a lower bound $L\left( N \right) \le Q_{WR}^\varepsilon \left( N \right)$ for the capacity with repeater (WR). If we find that $L\left( N \right)  >B_{DF}^\varepsilon \left( N \right)$, then we have successfully demonstrated that the quantum repeater improves our ability to transmit qubits over a direct transmission line.

### Usage to assess quantum error-correcting schemes

We note that in a similar way we can make statements about the performance of a QEC scheme for storing qubits with arbitrarily correlated errors. Suppose that we wish to compare how well an error-correcting scheme encoding one logical qubit using multiple physical qubits compares to using just one of the physical qubits directly. We can again employ the result of^8^ to first derive (a bound on) the one-shot quantum capacity $B_P^\varepsilon \left( N \right)$ if we used the physical qubit (*P*) *N* times with error *ε*. We then execute the capacity estimation protocol to estimate the capacity of the logical (LO) qubit channel $L\left( N \right) < Q_{{\rm{LO}}}^\varepsilon \left( N \right)$. If we find that $L\left( N \right)  >B_P^\varepsilon \left( N \right)$, then we can conclude that the logical qubit is an improvement for storing quantum information over one physical qubit.

### Other open questions

Our result assumes that the system on which the channel acts is composed of qubits. An interesting open question is whether this restriction can be removed and an analogous bound can be derived for channels of arbitrary dimension and composition.

It would also be interesting to see our bound extended to continuous variable systems. There are many tools already available^22–25^ that may be useful to perform such an analysis, but it remains to be determined how exactly they can be applied to such systems.

## Methods

To prove the bound on the one-shot quantum capacity, we combine several results. First, as we recapitulate in more detail in the Supplementary Note 1 through 4, it has been shown that the one-shot quantum capacity is bounded by the one-shot capacity of entanglement transmission $Q_{{\rm{ent}}}^\varepsilon \left( \Lambda \right)$ ^26^. More precisely, it holds that for every channel Λ and for every *ε* > 0^7^,

14 $${Q^\varepsilon }\left( \Lambda \right) \ge Q_{{\rm{ent}}}^{\varepsilon /2}\left( \Lambda \right) - 1.$$

The one-shot capacity of entanglement transmission, in turn, has been proved to be bounded by the smooth min-entropy $H_{{\rm{min}}}^\varepsilon {\left( {A{\rm{|}}E} \right)_\rho }$, which is defined by

15 $$H_{{\rm{min}}}^\varepsilon {\left( {A\left| B \right.} \right)_\rho }: = \mathop {{\max }}\limits_{\rho ' \in {B^\varepsilon }\left( \rho \right)} {H_{{\rm{min}}}}{\left( {A\left| B \right.} \right)_{\rho '}},$$

where

16 $${H_{{\rm{min}}}}{\left( {A\left| B \right.} \right)_\rho }: = \mathop {{\max }}\limits_{{\sigma _B}} \,{\rm{sup}}\left\{ {\lambda \in {\Bbb R}\left| {{\rho _{AB}}} \right. \le {2^{ - \lambda }}{I_A} \otimes {\sigma _B}} \right\}.$$

It has been shown that^7,8,27^

17 $$Q_{{\rm{ent}}}^\varepsilon \left( \Lambda \right) \ge \mathop {{\sup }}\limits_{\eta \in \left( {0,\sqrt \varepsilon } \right)} \left( {H_{{\rm{min}}}^{\sqrt \varepsilon - \eta }{{\left( {A\left| E \right.} \right)}_\rho } - 4{\rm{lo}}{{\rm{g}}_2}\frac{1}{\eta } - 1} \right),$$

Here, the smooth min-entropy is evaluated for the state

18 $${\rho _{AE}} = \left( {{I_A} \otimes \Lambda _{A' \to E}^c} \right)\left( {{\Phi _{AA'}}} \right),$$

where ${\Phi _{AA'}}$ is a maximally entangled state over the input system $A'$ and a copy *A* of it, and where $\Lambda _{A' \to E}^c$ is the complementary channel of the channel ${\Lambda _{A' \to B}}$. The system *E* is the environment of the channel (see refs. ^8,28^ and Supplementary Note 2 for more details). Taking together the results Eqs. (14) and (17), we get that for all *ε* > 0,

19 $${Q^\varepsilon }\left( \Lambda \right) \ge \mathop {{\sup }}\limits_{\eta \in \left( {0,\sqrt {\varepsilon /2} } \right)} \left( {H_{{\rm{min}}}^{\sqrt {\varepsilon /2} - \eta }{{\left( {A|E} \right)}_\rho } - 4\mathop {{\log }}\nolimits_2 \frac{1}{\eta } - 2} \right).$$

Therefore, the min-entropy bounds the one-shot quantum capacity.

Estimating the min-entropy has been a subject of intense research in quantum key distribution (QKD). However, min-entropy estimation protocols in QKD cannot be directly applied here, because they estimate the min-entropy $H_{{\rm{min}}}^\varepsilon \left( {X\left| E \right.} \right)$ for classical information *X*, while in the bound Eq. (17), the system *A* holds quantum information. We bridge this gap: as our main technical contribution, we show in the Supplementary Note 3 that for a system *A* that is composed of qubits, it holds that for every *ε* > 0 and every $\varepsilon ',\varepsilon '' \ge 0$,

20 $$H_{{\rm{min}}}^{3\varepsilon + \varepsilon ' + 4\varepsilon ''}{\left( {A\left| E \right.} \right)_\rho } \ge Nq - \left( {H_{{\rm{max}}}^{\varepsilon ''}{{\left( {X\left| B \right.} \right)}_\rho } + H_{{\rm{max}}}^{\varepsilon '}{{\left( {Z\left| B \right.} \right)}_\rho }} \right) - 2\,{\rm{lo}}{{\rm{g}}_2}\frac{2}{{{\varepsilon ^2}}}{\kern 1pt} .$$

Inequality Eq. (20) reduces estimating the min-entropy of quantum information *A* to estimating the max-entropy of measurement outcomes *X* and *Z* on the system *A*.

We prove inequality Eq. (20) using three main ingredients. First, we use an uncertainty relation for the smooth min- and max-entropies^29^. Second, we use a duality relation for the smooth min- and max-entropies^30,31^. These two ingredients were also used in modern security proofs of quantum key distribution^15^. We combine these two tools with a third tool, namely a chain rule theorem for the smooth max-entropy^32^ to arrive at the bound in inequality Eq. (20).

Given inequalities Eqs. (17) and (20), all we are left to do is to devise a protocol that estimates the max-entropies of *X* and *Z* given Bob’s quantum information *B*. Here we can make use of protocols in quantum key distribution that estimate exactly such a quantity. We show in the Supplementary Notes 4–6 how two such protocols (one for the max-entropy of *X* and one for the max-entropy of *Z*) can be combined into one protocol, which estimates both quantities simultaneously. The resulting protocol, which we presented in two versions, is given by Protocol 1 and Protocol 2 in the Results section. Our bound on the one-shot quantum capacity of the channel, inequality Eq. (9), is obtained by combining inequalities Eqs. (14) and (17) with these max-entropy estimation techniques.

### Data availability

The authors declare that all data supporting this study are contained within the article and its supplementary files.

## Electronic supplementary material

Supplementary Information
