# Supplementary material for: Capacity estimation and verification of quantum channels with arbitrarily correlated errors
Source: Nat Commun. 2018 Jan 2;9:27. doi: 10.1038/s41467-017-00961-2 (PMC5750239; doi:10.1038/s41467-017-00961-2)
Supplement: Supplementary file 1 — Supplementary Information [file 41467_2017_961_MOESM1_ESM.pdf]

Supplementary Note 1: Mathematical preliminaries and conventions

In supplementary note 1, we recapitulate some basic definitions. The material presented should not be seen as an introduction to the subject. We only state the definitions here to avoid ambiguity and to clarify our notation. The following definition clarifies our notation for operator spaces.

Definition 1 (Operator spaces): For a finite-dimensional complex inner product space $\mathcal{H}$, we define the following sets of operators on $\mathcal{H}$:

- $\mathrm{End}(\mathcal{H}) := \{L : \mathcal{H} \to \mathcal{H} \mid L \text{ linear}\}$, (endomorphisms on $\mathcal{H}$)
- $\mathrm{Herm}(\mathcal{H}) := \left\{L \in \mathrm{End}(\mathcal{H}) \mid L^\dagger = L\right\}$, (Hermitian operators on $\mathcal{H}$)
- $\mathcal{S}(\mathcal{H}) := \{\rho \in \mathrm{Herm}(\mathcal{H}) \mid \rho \geq 0, \mathrm{tr}(\rho) = 1\}$, (states / density operators on $\mathcal{H}$)
- $\mathcal{S}^{\leq}(\mathcal{H}) := \{\rho \in \mathrm{Herm}(\mathcal{H}) \mid \rho \geq 0, \mathrm{tr}(\rho) \leq 1\}$. (subnormalized states on $\mathcal{H}$)

Next, we make some general conventions.

Conventions : Throughout this document, we make use of the following conventions:

- log denotes the binary logarithm (base 2) and ln denotes the natural logarithm (base e).
- Quantum systems are assumed to be finite-dimensional, and the symbol $\mathcal{H}$ always denotes a finite-dimensional complex inner product space.
- A single subscript of $\mathcal{H}$ refers to the system associated with the space (for example, $\mathcal{H}_A$ is the space of system A).
- We use multiple subscripts of $\mathcal{H}$ to refer to a space of a joint system (for example, $\mathcal{H}_{AB} = \mathcal{H}_A \otimes \mathcal{H}_B$).
- For multipartite states, such as $\rho_{ABE} \in \mathcal{S}(\mathcal{H}_{ABE})$, we denote its reduced states by according changes of the subscript, e.g. $\rho_{AE} := \mathrm{tr}_B(\rho_{ABE})$, $\rho_A := \mathrm{tr}_B(\mathrm{tr}_E(\rho_{ABE}))$.

The formal definition of a quantum channel goes as follows.

Definition 2 (Quantum channel): Let A and B be quantum systems.

- The **identity channel** on A, denoted by I_A, is the linear map

$$\begin{array}{rccc} I_A: & \mathrm{End}(\mathcal{H}_A) & \to & \mathrm{End}(\mathcal{H}_A) \\ & \rho_A & \mapsto & \rho_A \,. \end{array} \tag{1}$$

- A **quantum channel** from A to B is a linear map

$$\Lambda : \mathrm{End}(\mathcal{H}_A) \to \mathrm{End}(\mathcal{H}_B) \tag{2}$$

which is trace-preserving, i.e.

$$\mathrm{tr}(\Lambda(\rho_A)) = \mathrm{tr}(\rho_A) \quad \forall\, \rho_A \in \mathrm{End}(\mathcal{H}_A)\,, \tag{3}$$

and which is completely positive. That is, for any quantum system E of any dimension $d_E \in \mathbb{N}_+$, the map $\Lambda \otimes I_E$ is a positive map,

$$(\Lambda \otimes I_E)(\rho_{AE}) \geq 0 \quad \forall\, \rho_{AE} \in \mathcal{H}_{AE}\,. \tag{4}$$

Such a map is called a **trace-preserving completely positive map** (abbreviated as **TPCPM**). (According to this definition, the terms "channel" and "TPCPM" are equivalent. In practice, the term channel is preferred when speaking of the evolution of a system in a physical sense, while the term TPCPM refers to the map as a mathematical object. However, this distinction is often not very strict.)

In order to define the the one-shot quantum capacity of a channel and the smooth entropies of quantum channels below, we need to make use of some distance measures.

Definition 3 (Distance measures): On the above operator spaces, we define the following distance measures:

- The **trace norm** on $\mathrm{End}(\mathcal{H})$ is defined as $\|L\|_1 = \mathrm{tr}\left(\sqrt{L^\dagger L}\right)$.
- The **trace distance** on $\mathrm{End}(\mathcal{H})$ is defined as $D(\rho,\sigma) := \frac{1}{2}\|\rho - \sigma\|_1$.
- The **generalized fidelity** [14] on $\mathcal{S}^{\leq}(\mathcal{H})$ is defined as $F(\rho,\sigma) := \left\|\sqrt{\rho}\sqrt{\sigma}\right\|_1 + \sqrt{(1-\mathrm{tr}\rho)(1-\mathrm{tr}\sigma)}$.

- The **fidelity** is given by the restriction of the generalized fidelity to $\mathcal{S}(\mathcal{H})$, resulting in $F(\rho,\sigma) = \left\|\sqrt{\rho}\sqrt{\sigma}\right\|$.
- The **purified distance** on $\mathcal{S}^{\leq}(\mathcal{H})$ is defined as $P(\rho,\sigma) := \sqrt{1 - F(\rho,\sigma)^2}$.
- The ε**-ball** around a subnormalized state $\rho \in \mathcal{S}^{\leq}(\mathcal{H})$ is given by $B^{\varepsilon}(\rho) := \{\rho' \in \mathcal{S}^{\leq}(\mathcal{H}) \mid P(\rho,\rho') \leq \varepsilon\}$.

Next, we define two kinds of one-shot capacities for quantum channels. There are (at least) two meaningful definitions of a capacity. In the i.i.d. scenario, these two capacities happen to coincide, and they are just referred to as the quantum capacity of a quantum channel. In the one-shot case, however, the two capacities are distinct, so it is not a priori clear which one should be chosen as the one-shot quantum capacity $Q^{\varepsilon}(\Lambda)$. Here, we follow Buscemi and Datta [2], identifying the one-shot capacity of minimum output fidelity as the one-shot quantum capacity. More precisely, we define the following.

Definition 4 (One-shot capacities): Let $\Lambda : \mathrm{End}(\mathcal{H}_A) \to \mathrm{End}(\mathcal{H}_B)$ be a quantum channel, let $\varepsilon \geq 0$.

- The **one-shot capacity of minimum output fidelity** $Q^{\varepsilon}(\Lambda)$ of the channel with respect to ε, which we also call the **one-shot quantum capacity** of the channel, is defined as

$$Q^{\varepsilon}(\Lambda) := \max\{\log m \mid F_{\min}(\Lambda, m) \geq 1 - \varepsilon\}\,, \tag{5}$$

where

$$F_{\min}(\Lambda, m) := \max_{\substack{\mathcal{H}'_A \subseteq \mathcal{H}_A \\ \dim\left(\mathcal{H}'_A\right) = m}} \max_{\mathcal{D}} \min_{|\phi\rangle \in \mathcal{H}'_A} \langle\phi|(\mathcal{D} \circ \Lambda)(\phi)|\phi\rangle\,. \tag{6}$$

The inner maximization ranges over all channels $\mathcal{D} : \mathrm{End}(\mathcal{H}_B) \to \mathrm{End}(\mathcal{H}_A)$ (decoding channels).

- The **one-shot capacity of entanglement transmission** $Q^{\varepsilon}_{\mathrm{ent}}(\Lambda)$ of the channel is defined as

$$Q^{\varepsilon}_{\mathrm{ent}}(\Lambda) := \max\{\log m \mid F_{\mathrm{ent}}(\Lambda, m) \geq 1 - \varepsilon\}\,, \tag{7}$$

where

$$F_{\mathrm{ent}}(\Lambda, m) := \max_{\substack{\mathcal{H}_M \subseteq \mathcal{H}_A \\ \dim(\mathcal{H}_M) = m}} \max_{\mathcal{D}} \langle\Phi_{MM'}|(\mathcal{D} \circ \Lambda)(\Phi_{MM'})|\Phi_{MM'}\rangle$$

The maximization over $\mathcal{D}$ is as above, and the state

$$|\Phi_{MM'}\rangle = \frac{1}{\sqrt{\dim(\mathcal{H}_M)}} \sum_{i=1}^{\dim(\mathcal{H}_M)} (|i\rangle_M \otimes |i\rangle_{M'}) \tag{8}$$

is a maximally entangled state on the subsystem $\mathcal{H}_M$ and a copy $\mathcal{H}_{M'}$ of it.

Although these two capacities are distinct, we will see below that they are comparable in the sense that they bound each other (see inequality Supplementary Equation 13 below). It is important to note that in Definition 4, we follow the definitions in [2] and [13] by using the fidelity as the figure of merit. Other sources, such as [5], define the one-shot capacities with the trace distance as the figure of merit. This will have consequences in Section 2, when a bound on $Q^{\varepsilon}_{\mathrm{ent}}(\Lambda)$ is converted from one definition to another (see inequality Supplementary Equation 20 below).

Now we define the (smooth) min- and max-entropy of a bipartite quantum state.

Definition 5 (Min- and max-entropy): Let $\rho_{AB} \in \mathcal{S}^{\leq}(\mathcal{H}_{AB})$ be a subnormalized bipartite state.

- The **min-entropy** of A conditioned on B for the state ρ_{AB} is defined [9] as

$$H_{\min}(A|B)_{\rho} := \max_{\sigma_B \in \mathcal{S}^{\leq}(\mathcal{H}_B)} \sup\{\lambda \in \mathbb{R} \mid \rho_{AB} \leq 2^{-\lambda} I_A \otimes \sigma_B\}\,. \tag{9}$$

- The **max-entropy** of A given B for the state ρ_{AB} is defined [4] as

$$H_{\max}(A|B)_{\rho} := \max_{\sigma_B \in \mathcal{S}^{\leq}(\mathcal{H}_B)} \log \left\|\sqrt{\rho_{AB}}\sqrt{I_A \otimes \sigma_B}\right\|_1^2\,. \tag{10}$$

Definition 6 (Smooth min- and max-entropy): Let $\rho_{AB} \in \mathcal{S}^{\leq}(\mathcal{H}_{AB})$ be a bipartite state and let $\varepsilon \geq 0$.

- The ε**-smooth max-entropy** of A conditioned on B is defined as

$$H^{\varepsilon}_{\max}(A|B)_{\rho} := \min_{\rho' \in B^{\varepsilon}(\rho)} H_{\max}(A|B)_{\rho'}\,, \tag{11}$$

- The ε**-smooth min-entropy** of A conditioned on B is defined as

$$H^{\varepsilon}_{\min}(A|B)_\rho := \max_{\rho' \in B^{\varepsilon}(\rho)} H_{\min}(A|B)_{\rho'} \,. \tag{12}$$

For states that are defined on more systems than labeled in the entropy, the entropy is evaluated for the according reduced state. For example, given a state $\rho_{ABE} \in \mathcal{H}_{ABE}$, the smooth min-entropy $H^{\varepsilon}_{\min}(A|E)_\rho$ is evaluated for $\rho_{AE} = \mathrm{tr}_B(\rho_{ABE})$.

To avoid confusion with other sources that define the smooth min- and max-entropies, it is important to note two things.

- Firstly, the max-entropy, as we defined it in Definition 5, coincides with the Rényi entropy of order 1/2, whereas in some older sources, it was defined as the Rényi entropy of order 0 [9].
- Secondly, the smooth entropies, as we defined them in Definition 6, measure the distance in the purified distance, whereas in some older sources, it was defined with respect to the trace distance [9].

There are several reasons for making the definitions as we use them here. One important reason is that this way, the smooth min- and max-entropies satisfy a duality relation [14] that we will exploit in Section 3 (see Lemma 9).

Supplementary Note 2: Background: Proof of the min-entropy bound on the one-shot quantum capacity

In supplementary note 2, we explain the details of the min-entropy bound on the one-shot quantum capacity (inequality (17) of the main article) and show how it is derived. This is not a new result, but an application of results that are well-established in quantum information science, which we provide here for convenience of the reader.

As mentioned in the main article, as the first step in the derivation of inequality (17), we note that the one-shot quantum capacity $Q^{\varepsilon}(\Lambda)$ of a quantum channel Λ can be lower-bounded by the one-shot capacity of entanglement transmission $Q^{\varepsilon}_{\mathrm{ent}}(\Lambda)$. More precisely, Barnum, Knill and Nielsen [1] have shown that (here we use the form presented in [2])

$$\forall\, \varepsilon > 0 : Q^{\varepsilon}_{\mathrm{ent}}(\Lambda) - 1 \leq Q^{2\varepsilon}(\Lambda) \leq Q^{4\varepsilon}_{\mathrm{ent}}(\Lambda) \,. \tag{13}$$

In particular,

$$\forall\, \varepsilon > 0 : \quad Q^{\varepsilon}(\Lambda) \geq Q^{\varepsilon/2}_{\mathrm{ent}}(\Lambda) - 1 \,. \tag{14}$$

In the next step, we will bound $Q^{\varepsilon}_{\mathrm{ent}}(\Lambda)$. Before we do that, it is helpful to extend our picture with the Stinespring dilation of the channel and a purification of the input state, as shown in Supplementary Figures 1 and 2. Readers who are already familiar with these concepts may skip this part and continue reading below Supplementary Figure 2.

Recapitulate the situation that we consider: we are given a quantum channel Λ that takes a quantum system on Alice's side as its input and outputs another quantum system on Bob's side. It is helpful to give these input and output systems their own labels. We denote the input system on Alice's side by A' and the output system on Bob's side by B (the reason for choosing A' instead of A will become clear below). The situation is depicted in Supplementary Figure 1 (left).

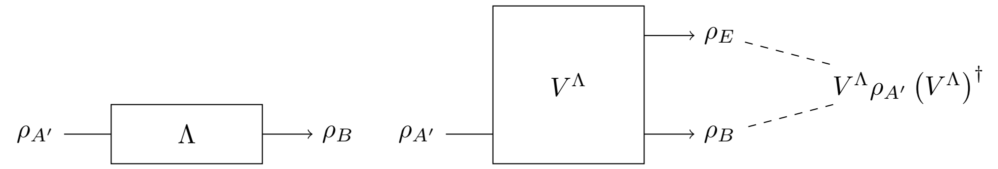

Supplementary Figure 1: An arbitrary quantum channel Λ and its Stinespring dilation V^{Λ}. Left: A channel Λ from A' to B. Right: Stinespring dilation of Λ from A' to BE. For every channel Λ from A' to B (left), there is an isometry V^{Λ} (right) such that $\Lambda(\rho_{A'}) = \mathrm{tr}(V^{\Lambda}\rho_{A'}(V^{\Lambda})^{\dagger})$. Isometries have the important property that they map pure input states to pure output states. This will be important below, where we purify the input state $\rho_{A'}$ (see Supplementary Figure 2).

For our purposes, it is useful to extend this picture. Mathematically speaking, a quantum channel is a trace-preserving completely positive map that maps density operators $\rho_{A'}$ to density operators ρ_B,

$$\Lambda : \mathcal{S}(\mathcal{H}_{A'}) \to \mathcal{S}(\mathcal{H}_B) \,. \tag{15}$$

The Stinespring dilation theorem [11] states that for every such completely positive map Λ, there is a system E of dimension $d_E \leq d_{A'}^2$ and a linear isometry

$$V^\Lambda : \mathcal{H}_{A'} \to \mathcal{H}_B \otimes \mathcal{H}_E \tag{16}$$

such that

$$\Lambda(\rho_{A'}) = \mathrm{tr}_E \left(V^\Lambda \rho_{A'} \left(V^\Lambda\right)^\dagger \right) . \tag{17}$$

The extended picture is shown in Supplementary Figure 1 (right). The map V^Λ is an isometric extension or Stinespring dilation of the channel Λ, and is a standard tool in quantum information theory [18].

We extend this picture further using another standard tool in quantum information. The input state $\rho_{A'}$ of the channel may not be a pure state but a mixed state. This is inconvenient, as many useful mathematical statements require the involved states to be pure. However, we can work around this by considering a purification of $\rho_{A'}$, that is, a system A of dimension $d_A \leq d_{A'}$ and a pure state $\rho_{AA'}$ such that $\mathrm{tr}_A(\rho_{AA'}) = \rho'_A$. Every state has such a purification, but it is not unique [6]. Here, for every state $\rho_{A'}$, we consider a purification with $d_A = d_{A'}$, which is called the canonical purification $\psi^\rho_{AA'}$, and is given by $\psi^\rho_{AA'} = |\psi^\rho\rangle\langle\psi^\rho|_{AA'}$, where

$$|\psi^\rho\rangle_{AA'} = d_{A'} \ \left(I_A \otimes \sqrt{\rho_{A'}}\right) |\Phi\rangle_{AA'} . \tag{18}$$

Here, $|\Phi_{AA'}\rangle$ is the maximally entangled basis with respect to some bases $\{|i\rangle_A\}_i$ and $\{|i\rangle_{A'}\}_i$ for $\mathcal{H}_A$ and $\mathcal{H}_{A'}$, respectively (their choice is irrelevant for what we consider),

$$|\Phi\rangle_{AA'} = \sum_{i=1}^{d_{A'}} \frac{1}{\sqrt{d_{A'}}} |i\rangle_A \otimes |i\rangle_{A'} . \tag{19}$$

By extending system A' to system AA' in this way, we arrive at the overall picture shown in Supplementary Figure 2. After the channel Λ acted on system A', we not only consider the output system B but the tripartite system ABE, which is in a state ρ_{ABE}. Since isometries map pure input states to pure output states, is is a pure state. This is the reason for this extension. It will allow us to apply the duality relation in Section 3 (see Lemma 9).

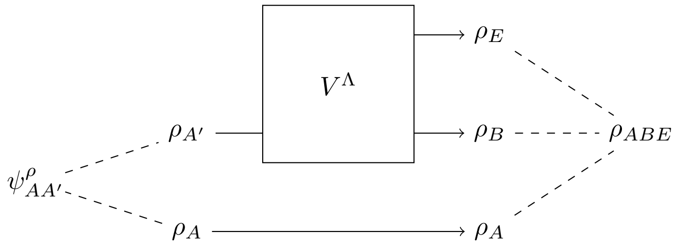

Supplementary Figure 2: The fully purified diagram for a quantum channel. A quantum channel taking input A' and mapping it to output B can be purified. By taking the environment E suitably large, the overall state is always pure: Since the input state $\psi^\rho_{AA'}$ is pure and $I_A \otimes V^\Lambda$ is an isometry, the output state ρ_{ABE} is pure.

Now we are ready to proceed with the next step in the derivation of inequality (17). It turns out that the one-shot capacity of entanglement transmission $Q^\varepsilon_{\mathrm{ent}}(\Lambda)$ of the channel Λ can be bounded by functions of the state ρ_{ABE} that we described above. Buscemi and Datta [2] have shown that a lower bound on the one-shot capacity of entanglement transmission can be formulated in terms of a maximization of an entropic quantity. Subsequently, Morgan and Winter tightened this bound and translated it to an optimization of the smooth min-entropy of the state $\rho_{AE} = \mathrm{tr}_B(\rho_{ABE})$ [5]. Here we use this bound in the following form [13]:

$$\forall\, \varepsilon > 0 : \quad Q^\varepsilon_{\mathrm{ent}}(\Lambda) \geq \sup_{\eta \in (0,\sqrt{\varepsilon})} \ \sup_{\rho_{A'} \in \mathcal{S}(\mathcal{H}_{A'})} \left(H^{\sqrt{\varepsilon}-\eta}_{\min}(A|E)_\rho - 4 \log \frac{1}{\eta} - 1 \right) . \tag{20}$$

The square root in the smoothing parameter is a consequence of the fact that the bound Supplementary Equation 20 was derived through conversion from a bound where the figure of merit for entanglement transmission was the purified distance [5] instead of the fidelity [13].

Next, we drop the maximization over the state $\rho_{A'} \in \mathcal{S}(\mathcal{H}_{A'})$ by choosing the maximally mixed state $\rho_{A'} = I_{A'}/d_{A'}$. This way, we arrive at another lower bound:

$$\forall\, \varepsilon > 0 : \quad Q^\varepsilon_{\mathrm{ent}}(\Lambda) \geq \sup_{\eta \in (0,\sqrt{\varepsilon})} \left(H^{\sqrt{\varepsilon}-\eta}_{\min}(A|E)_\rho - 4 \log \frac{1}{\eta} - 1 \right) . \tag{21}$$

This corresponds to the case where the input state $\psi^{\rho}_{AA'}$ in Supplementary Figure 2 is given by the maximally entangled state $\Phi_{AA'}$ (see Supplementary Equation 18 and Supplementary Equation 19 above). This is of particular importance for us because we can actually estimate $H^{\varepsilon}_{\min}(A|E)$ in that case (see Section 4). Combining inequalities Supplementary Equation 14 and Supplementary Equation 21, we get the bound

$$\forall\, \varepsilon > 0 : \quad Q^{\varepsilon}(\Lambda) \geq \sup_{\eta \in (0, \sqrt{\varepsilon/2})} \left(H^{\sqrt{\varepsilon/2}-\eta}_{\min}(A|E)_{\rho} - 4 \log \frac{1}{\eta} - 2 \right) . \tag{22}$$

Supplementary Note 3: Proof of the bound on the min-entropy

In Section 2, we have seen that the one-shot quantum capacity $Q^{\varepsilon}(\Lambda)$ can be bounded in terms of the smooth min-entropy $H^{\varepsilon}_{\min}(A|E)$ of an appropriately defined state ρ_{AE}. In this supplementary note, we prove that this min-entropy is bounded by the smooth max-entropies $H^{\varepsilon}_{\max}(X|B)$ and $H^{\varepsilon}_{\max}(Z|B)$ of measurement X and Z on A. More precisely, we will show:

$$\forall\, \varepsilon > 0, \forall\, \varepsilon', \varepsilon'' \geq 0 : \quad H^{3\varepsilon+\varepsilon'+4\varepsilon''}_{\min}(A|E)_{\rho} \geq Nq - \left(H^{\varepsilon''}_{\max}(X|B)_{\rho} + H^{\varepsilon'}_{\max}(Z|B)_{\rho} \right) - 2 \log \frac{2}{\varepsilon^2} \tag{23}$$

(see Theorem 13 below). Using this inequality, we will prove our bound on the one-shot quantum capacity in terms of the protocol parameters in Section 4.

In the following, we will cite some lemmas that we will need for the proof of the bound Supplementary Equation 23. The most important ones are:

- an uncertainty relation for the smooth min- and max-entropies [16],
- a chain rule theorem for the smooth max-entropy [17] and
- a duality relation for the smooth min- and max-entropies [4, 14].

We start with the uncertainty relation for the smooth min- and max-entropies.

Lemma 7 (Smooth min-max uncertainty): Let $\rho_{ABE} \in \mathcal{S}(\mathcal{H}_{ABE})$ be a pure tripartite state where A is an N-qubit system, let $X = \{X_0, X_1\}$ and $Z = \{Z_0, Z_1\}$ be qubit POVMs. Consider the states ρ_{XBE} and ρ_{ZBE} that arise from measuring all of the N qubits of system A with respect to X and Z, respectively, and storing the outcomes in a classical register X and Z, respectively,

$$\rho_{XBE} = \sum_{x \in \{0,1\}^n} P_X(x)\ |x\rangle\langle x| \otimes \rho^{x}_{BE} , \tag{24}$$

$$\rho_{ZBE} = \sum_{z \in \{0,1\}^n} P_Z(z)\ |z\rangle\langle z| \otimes \rho^{z}_{BE} . \tag{25}$$

where

$$P_X(x) = \mathrm{tr}\left(\Pi_X(x) \rho_A\right) , \tag{26}$$

$$\Pi_X(x) = \bigotimes_{i=1}^{n} X_{x_i} \quad \text{for } x = (x_1, \ldots, x_n) \in \{0,1\}^n , \tag{27}$$

$$\rho^{x}_{BE} = \frac{\mathrm{tr}_A((\Pi_X(x) \otimes I_{BE}) \rho_{ABE} (\Pi_X(x) \otimes I_{BE}))}{P_X(x)} , \tag{28}$$

and analogously for ρ_{ZBE}. Then for $\varepsilon \geq 0$,

$$H^{\varepsilon}_{\min}(X|E)_{\rho} + H^{\varepsilon}_{\max}(Z|B)_{\rho} \geq Nq , \tag{29}$$

where

$$q = -\log \max_{i,j} \left\| \sqrt{X_i} \sqrt{Z_j} \right\|^2_{\infty} . \tag{30}$$

The parameter q is the **preparation quality**. If X and Z are measurements with respect to mutually unbiased bases, then $q = 1$.

The chain rule that we will use is actually just one out of a series of chain rule inequalities proved in [17]. The particular form that we use here can be found in [12].

Lemma 8 (Chain rule for smooth max-entropy): Let $\rho_{ABC} \in \mathcal{S}^{\leq}(\mathcal{H}_{ABC})$ be a tripartite state, let $\varepsilon > 0$, $\varepsilon' \geq 0$, $\varepsilon'' \geq 0$. Then

$$H^{\varepsilon+\varepsilon'+2\varepsilon''}_{\max}(AB|C)_{\rho} \leq H^{\varepsilon'}_{\max}(A|BC)_{\rho} + H^{\varepsilon''}_{\max}(B|C)_{\rho} + \log \frac{2}{\varepsilon^2} . \tag{31}$$

The duality relation between the smooth min- and max-entropy, or min-max duality, for short, relates the smooth min-entropy of a state to the max-entropy of a purification of the state. It was first proved for the unsmoothed min- and max-entropy König, Renner and Schaffner in [4]. The min-max duality for the smooth entropies is due to Tomamichel, Colbeck and Renner [14].

Lemma 9 (Min-max duality): Let $\rho_{ABE} \in \mathcal{S}(\mathcal{H}_{ABE})$ be a pure tripartite state, let $\varepsilon \geq 0$. Then

$$H_{\min}(A|E)_\rho = -H_{\max}(A|B)_\rho \quad \text{and} \tag{32}$$

$$H^\varepsilon_{\min}(A|E)_\rho = -H^\varepsilon_{\max}(A|B)_\rho \,. \tag{33}$$

Apart from these three main ingredients, we will also make use of three smaller lemmas. The first one states that the smooth min- and max-entropies are invariant under isometries [12].

Lemma 10 (Invariance under isometries): Let $\rho_{AB} \in \mathcal{S}^{\leq}(\mathcal{H}_{AB})$ be a bipartite state, let $\varepsilon \geq 0$. Then for all isometries $V : \mathcal{H}_A \to \mathcal{H}_{A'}$ and $W : \mathcal{H}_B \to \mathcal{H}_{B'}$, the embedded state $\sigma_{A'B'} = (V \otimes W)\rho_{AB}(V^\dagger \otimes W^\dagger)$ satisfies

$$H^\varepsilon_{\min}(A|B)_\rho = H^\varepsilon_{\min}(A'|B')_\sigma \quad \text{and} \quad H^\varepsilon_{\max}(A|B)_\rho = H^\varepsilon_{\max}(A'|B')_\sigma \,. \tag{34}$$

In simple terms, the following lemma states that "forgetting" side information cannot decrease one's uncertainty. It is a special case of a more general theorem, called the data processing inequality [12]. We only state the more special case that we are interested in.

Lemma 11 : Let $\rho_{ABC} \in \mathcal{S}^{\leq}(\mathcal{H}_{ABC})$ be a tripartite state. Then

$$H_{\max}(A|BC) \leq H_{\max}(A|B) \,. \tag{35}$$

Finally, the last lemma that we add to our list of tools shows how the (unsmoothed) max-entropy simplifies in the case where classical side information is given.

Lemma 12 : Let $\rho_{ACX} \in \mathcal{S}^{\leq}(\mathcal{H}_{ACX})$ be a state of the form

$$\rho_{ACX} = \sum_x p_x \, \rho^x_{AC} \otimes |x\rangle\langle x|_X \,, \quad \text{where} \quad \rho^x_{AC} \in \mathcal{S}^{\leq}(\mathcal{H}_{AC}) \,. \tag{36}$$

Then [12]

$$H_{\max}(A|CX)_\rho = \log\left(\sum_x P_X(x) \; 2^{H_{\max}(A|C)_{\rho^x_{AC}}}\right) \,. \tag{37}$$

Now we are ready to state the theorem formally and prove it.

Theorem 13 : Let $\rho_{ABE} \in \mathcal{S}(\mathcal{H}_{ABE})$ be a pure tripartite state where A and B are each an N-qubit system, let $X = \{X_0, X_1\}$ and $Z = \{Z_0, Z_1\}$ be non-trivial projective measurements on a qubit (that is, both elements are one-dimensional projectors). Consider the states ρ_{XBE} and ρ_{ZBE} that arise from measuring all of the N qubits of system A with respect to X and Z (as in Lemma 7). Then, for $\varepsilon > 0$ and $\varepsilon', \varepsilon'' \geq 0$, it holds that

$$H^{3\varepsilon+\varepsilon'+4\varepsilon''}_{\min}(A|E)_\rho \geq Nq - (H^{\varepsilon'}_{\max}(Z|B)_\rho + H^{\varepsilon''}_{\max}(X|B)_\rho) - 2\log\frac{2}{\varepsilon^2} \,, \tag{38}$$

where q is the preparation quality (as in Lemma 7).

Proof. Starting from ρ_{ABE}, we construct a purification $\rho_{AXX'BE}$ of ρ_{XBE}. Further below, we will expand the smooth max-entropy of this state using the chain rule (Lemma 8). Reformulating the terms in that expansion will lead us to the desired result.

Consider the product POVM elements

$$\Pi_X(x) = \bigotimes_{i=1}^N X_{x_i} \quad \text{for } x = (x_i)_{i=1}^N \in \{0,1\}^N \,. \tag{39}$$

We construct $\rho_{AXX'BE}$ from ρ_{ABE} by performing a coherent measurement on the A system with respect to the POVM formed by the elements Supplementary Equation 39. The outcome of this measurement is stored in two copies X and X' of a classical register. For $x \in \{0,1\}^N$, let V_x be the map

$$\begin{aligned} V_x : \quad \mathcal{H}_A \quad &\to \quad \mathcal{H}_{AXX'} \\ |\psi\rangle \quad &\mapsto \quad \Pi_X(x)|\psi\rangle \otimes |x\rangle_X \otimes |x\rangle_{X'} \,. \end{aligned} \tag{40}$$

We define the state $\rho_{AXX'BE} := V(\rho_{ABE})$, where

$$\begin{aligned} V : \quad \mathrm{End}(\mathcal{H}_{ABE}) \quad &\to \quad \mathrm{End}(\mathcal{H}_{AXX'BE}) \\ \rho_{ABE} \quad &\mapsto \quad \textstyle\sum_x (V_x \otimes I_{BE})\rho_{ABE}(V_x^\dagger \otimes I_{BE}) \,. \end{aligned} \tag{41}$$

The map V is an isometry that maps the pure state ρ_{ABE} to the pure state $\rho_{AXX'BE}$. Thus, by virtue of Lemma 9, it holds that

$$H_{\min}^{\varepsilon'}(X|E)_\rho = -H_{\max}^{\varepsilon'}(X|AX'B)_\rho \,. \tag{42}$$

We will use Supplementary Equation 42 further below.

Now we expand the max-entropy of $\rho_{AXX'BE}$ using the chain rule, Lemma 8:

$$H_{\max}^{\varepsilon+\varepsilon'+2(\varepsilon+2\varepsilon'')}(AXX'|B)_\rho \leq H_{\max}^{\varepsilon'}(X|AX'B)_\rho + H_{\max}^{\varepsilon+2\varepsilon''}(AX'|B)_\rho + \log\frac{2}{\varepsilon^2}\,. \tag{43}$$

The states ρ_{AB} and $\rho_{AXX'B}$ only differ by an isometry, so by Lemma 10, we have

$$H_{\max}^{\varepsilon+\varepsilon'+2(\varepsilon+2\varepsilon'')}(AXX'|B)_\rho = H_{\max}^{3\varepsilon+\varepsilon'+4\varepsilon''}(A|B)_\rho\,. \tag{44}$$

(It will become clear further below why we choose the smoothing parameter on the left hand side this way.) Moreover, the marginals $\rho_{AX'B}$ and ρ_{AXB} only differ by a unitary $\mathcal{H}_X \to \mathcal{H}_{X'}$ and therefore

$$H_{\max}^{\varepsilon+2\varepsilon''}(AX'|B)_\rho = H_{\max}^{\varepsilon+2\varepsilon''}(AX|B)_\rho \tag{45}$$

Combining Supplementary Equation 43, Supplementary Equation 44,and Supplementary Equation 45 yields

$$H_{\max}^{\varepsilon'}(X|AX'B)_\rho \geq H_{\max}^{3\varepsilon+\varepsilon'+4\varepsilon''}(A|B)_\rho - H_{\max}^{\varepsilon+2\varepsilon''}(AX|B)_\rho - \log\frac{2}{\varepsilon^2}\,. \tag{46}$$

Now we expand the term $H_{\max}^{\varepsilon+2\varepsilon''}(AX|B)$ using the chain rule:

$$H_{\max}^{\varepsilon+2\varepsilon''}(AX|B)_\rho \leq \underbrace{H_{\max}^{0}(A|XB)_\rho}_{H_{\max}(A|XB)_\rho} + H_{\max}^{\varepsilon''}(X|B)_\rho + \log\frac{2}{\varepsilon^2}\,. \tag{47}$$

Combining Supplementary Equation 46 with Supplementary Equation 47 allows us to infer

$$H_{\max}^{\varepsilon'}(X|AX'B)_\rho \geq H_{\max}^{3\varepsilon+\varepsilon'+4\varepsilon''}(A|B)_\rho - H_{\max}(A|XB)_\rho - H_{\max}^{\varepsilon''}(X|B)_\rho - 2\log\frac{2}{\varepsilon^2}\,. \tag{48}$$

Now we use Supplementary Equation 42 that we derived above to rewrite inequality Supplementary Equation 48 as

$$H_{\min}^{\varepsilon'}(X|E)_\rho \leq -H_{\max}^{3\varepsilon+\varepsilon'+4\varepsilon''}(A|B)_\rho + H_{\max}(A|XB)_\rho + H_{\max}^{\varepsilon''}(X|B)_\rho + 2\log\frac{2}{\varepsilon^2}\,. \tag{49}$$

Reordering terms and using Lemma 11 and the uncertainty relation for the smooth min- and max-entropy (Lemma 7), we get

$$H_{\max}^{3\varepsilon+\varepsilon'+4\varepsilon''}(A|B)_\rho \leq \underbrace{H_{\max}(A|XB)_\rho}_{\leq H_{\max}(A|X)_\rho} + H_{\max}^{\varepsilon''}(X|B)_\rho \underbrace{-H_{\min}^{\varepsilon'}(X|E)_\rho}_{\leq H_{\max}^{\varepsilon'}(Z|B)_\rho - Nq} + 2\log\frac{2}{\varepsilon^2} \tag{50}$$

$$\leq H_{\max}(A|X)_\rho + H_{\max}^{\varepsilon''}(X|B)_\rho + H_{\max}^{\varepsilon'}(Z|B)_\rho - Nq + 2\log\frac{2}{\varepsilon^2}\,. \tag{51}$$

Applying the duality relation (Lemma 9) to the left hand side of Supplementary Equation 51, we get

$$H_{\min}^{3\varepsilon+\varepsilon'+4\varepsilon''}(A|E)_\rho \geq Nq - H_{\max}(A|X)_\rho - \left(H_{\max}^{\varepsilon''}(X|B)_\rho + H_{\max}^{\varepsilon'}(Z|B)_\rho\right) - 2\log\frac{2}{\varepsilon^2}\,. \tag{52}$$

We are left to show that $H_{\max}(A|X)_\rho$ is upper bounded by 0. We show, more precisely, that $H_{\max}(A|X)_\rho = 0$. This goes as follows.

$$\begin{aligned}
\rho_{AX} &= \mathrm{tr}_{X'BE}(\rho_{AXX'BE}) && (53)\\
&= \mathrm{tr}_{X'BE}\left(\sum_x (V_x \otimes I_{BE})\rho_{ABE}(V_x^\dagger \otimes I_{BE})\right) && (54)\\
&= \mathrm{tr}_{X'}\left(\sum_x V_x \rho_A V_x^\dagger\right) && (55)\\
&= \sum_x \Pi_X(x)\rho_A \Pi_X(x) \otimes |x\rangle\langle x|_X && (56)\\
&= \sum_x P_X(x)\ \rho_A^x \otimes |x\rangle\langle x|_X\,, && (57)
\end{aligned}$$

where

$$P_X(x) = \mathrm{tr}(\Pi_X(x)\rho_A)\,, \tag{58}$$

$$\rho_A^x = \frac{\Pi_X(x)\rho_A\Pi_X(x)}{P_X(x)}\,. \tag{59}$$

Now we can apply Lemma 12 to Supplementary Equation 57: By setting the system C in the lemma to a trivial system ($\mathcal{H}_C \simeq \mathbb{C}$), we can deduce that

$$H_{\max}(A|X)_\rho = \log\left(\sum_x P_X(x)\; 2^{H_{\max}(A)_{\rho_A^x}}\right)\,, \tag{60}$$

where $H_{\max}(A)_{\rho_A^x}$ reduces to the unconditional form of the max-entropy,

$$H_{\max}(A)_{\rho_A^x} = \log\left\|\sqrt{\rho_A^x}\right\|_1^2 = \log\left(\mathrm{tr}\left(\sqrt{\rho_A^x}\right)\right)^2\,. \tag{61}$$

Since the $\Pi_X(x)$ are one-dimensional projectors, we have that

$$H_{\max}(A)_{\rho_A^x} = 0 \quad \text{for all } x \in \{0,1\}^N \tag{62}$$

and therefore $H_{\max}(A|X)_\rho = 0$, as claimed. Thus, we have proved that

$$H_{\min}^{3\varepsilon+\varepsilon'+4\varepsilon''}(A|E)_\rho \geq Nq - \left(H_{\max}^{\varepsilon''}(X|B)_\rho + H_{\max}^{\varepsilon'}(Z|B)_\rho\right) - 2\log\frac{2}{\varepsilon^2}\,, \tag{63}$$

which is what we wanted to show. □

Supplementary Note 4: Comparison to min-entropy estimation in QKD

In Section 2, we have seen that the one-shot quantum capacity of a channel is bounded by the min-entropy. In the last section, we have seen how the smooth min-entropy $H_{\min}^{3\varepsilon+\varepsilon'+4\varepsilon''}(A|E)$ can be bounded in terms of the max-entropy $H_{\max}^{\varepsilon''}(X|B)$ and $H_{\max}^{\varepsilon'}(Z|B)$ of the classical measurement outcomes X and Z on A. This puts us in a very good position, because we already know from quantum key distribution how to bound these max-entropies: a modern approach to quantum key distribution based on smooth entropies proves security by bounding exactly such a quantity.

In that approach, a QKD protocol is devised in which after sifting, Alice and Bob have n systems where they both measured in the X-basis and k systems where they both measured in the Z-basis. Then they exchange their outcomes in the Z-basis and determine the error rate λ. If the error rate λ does not exceed a specified error tolerance e_z, then they conclude that [15, 8, 7]

$$H_{\max}^{\varepsilon'}(Z|B)_\rho \leq nh(e_z + \mu(\varepsilon))\,, \tag{64}$$

where h denotes the binary entropy function and

$$\varepsilon' = \frac{\varepsilon}{\sqrt{p_{\mathrm{pass}}}}\,, \quad \mu(\varepsilon) = \sqrt{\frac{n+k}{nk}\frac{k+1}{k}\ln\frac{1}{\varepsilon}}\,. \tag{65}$$

Here p_{pass} is the probability that the correlation test (which checks whether $\lambda \leq e_z$) is passed, where $p = 1 - p_{\mathrm{pass}}$ is the parameter given in the theorem. The state ρ in inequality Supplementary Equation 64 is the state of the n qubits that have actually been measured in X. This means that from the error rate λ in one part of the qubits, one can infer a bound on $H_{\max}^{\varepsilon'}(Z|B)$ for the other part of the qubits. This is illustrated in Supplementary Figure 3.

In the QKD scenario we just described, the goal was to infer a bound on $H_{\max}^{\varepsilon'}(Z|B)$ on only a part of the total system from the error rate λ on its complement. In our one-shot quantum capacity estimation and verification protocols, the situation is a bit different. It is easier to discuss the verification protocol first, because it is conceptually closer to the QKD protocol from which we adopt the estimation techniques.

Supplementary Note 5: Proof for the verification protocol

In the verification protocol, the qubits are divided into three subsets: one subset of test qubits that are measured in the X-basis, one subset of test qubits that are measured in the Z-basis, and the data qubits that are not measured at all (see the top part of Supplementary Figure 4). In the main article, we stated the protocol such that each of theses three subets has the same size N. Here, we consider the more general case where each of theses subsets might have a different size $n, k, N \in \mathbb{N}_+$, respectively, and later specialize the result to $n = k = N$. This also helps

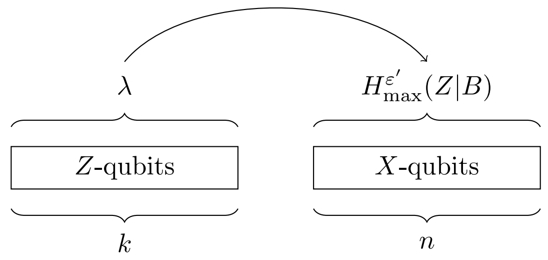

Supplementary Figure 3: Bounding the max-entropy from an error rate on a different part. In the QKD protocol that we consider [15, 8, 7], the test qubits are measured in Z and the key qubits are measured in X. For the security of the protocol, $H_{\max}^{\varepsilon'}(Z|B)$ needs to be bounded for the key qubits. This bound can be inferred from the error rate λ on the test qubits.

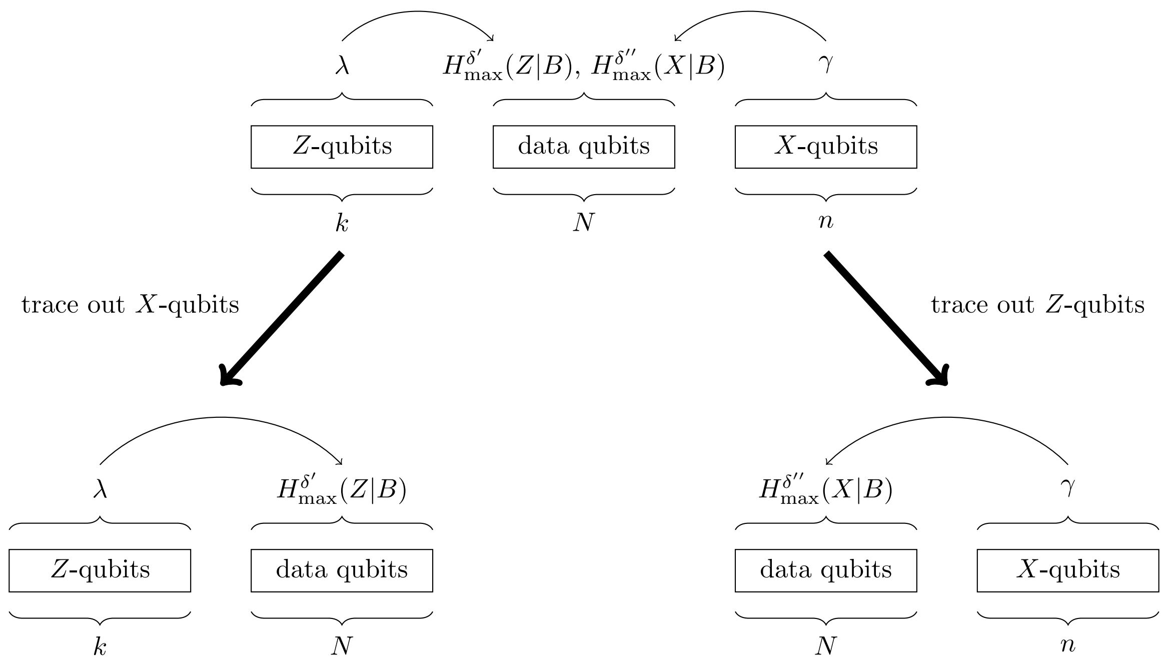

Supplementary Figure 4: Inference of the max-entropies in the verification protocol. Our verification protocol can be seen as the parallel execution of two QKD estimation protocols (see Supplementary Figure 3). Thus, if both $\gamma \leq e_x$ and $\lambda \leq e_z$, we get two bounds of the form Supplementary Equation 64.

us in the proof to keep track of which number we mean. Here, we denote the smoothing parameter by δ instead of ε (it will become clear below why it is useful to do so).

This situation may look more complicated than in the QKD scenario above. However, it turns out that our verification protocol can be seen as running the above QKD estimation two times in parallel (see the bottom part of Supplementary Figure 4). When we trace out the X-qubits, the remainder is in the same situation as in the QKD case as shown in Supplementary Figure 3, with the N data qubits taking the role of the qubits for which we bound the max-entropy $H_{\max}^{\delta'}(Z|B)$. Therefore, if we find that the error rate λ is below a tolerated error rate e_z, we conclude that

$$H_{\max}^{\delta'}(Z|B) \leq Nh(e_z + \mu_z(\delta))\,, \tag{66}$$

where

$$\delta' = \frac{\delta}{\sqrt{p_{\text{pass}}^z}}\,, \quad \mu_z(\delta) = \sqrt{\frac{N+k}{Nk}\frac{k+1}{k}\ln\frac{1}{\delta}} \tag{67}$$

and where p_{pass}^z is the probability that $\lambda \leq e_z$.

Likewise, when we trace out the Z-qubits, the remainder looks like in the QKD case, with the X-basis taking the role of the Z-basis and with the data qubits taking the role of the qubits for which we bound the max-entropy $H_{\max}^{\delta''}(X|B)$. If we find that the error rate γ is below a tolerated error rate e_x, then

$$H_{\max}^{\delta''}(X|B) \leq Nh(e_x + \mu_x(\delta))\,, \tag{68}$$

where

$$\delta'' = \frac{\delta}{\sqrt{p_{\text{pass}}^x}}, \quad \mu_x(\delta) = \sqrt{\frac{N+n}{Nn}\frac{n+1}{n}\ln\frac{1}{\delta}} \tag{69}$$

and where p_{pass}^x is the probability that $\gamma \le e_x$.

According to our verification protocol (see Protocol 2 in the main article), we are interested in the case where both $\gamma \le e_x$ and $\lambda \le e_z$. In that case, we can conclude that

$$H_{\max}^{\delta'}(Z|B) + H_{\max}^{\delta''}(X|B) \le N\Big(h(e_z + \mu_z(\delta)) + h(e_x + \mu_x(\delta))\Big)\,. \tag{70}$$

At this point, we can connect this bound with the bound that we derived in Section 3 (see Theorem 13), which says that

$$H_{\min}^{3\delta+\delta'+4\delta''}(A|E)_\rho \ge Nq - (H_{\max}^{\delta'}(Z|B)_\rho + H_{\max}^{\delta''}(X|B)_\rho) - 2\log\frac{2}{\delta^2}\,. \tag{71}$$

Combining inequalities Supplementary Equation 70 and Supplementary Equation 71, we get that

$$H_{\min}^{3\delta+\delta'+4\delta''}(A|E)_\rho \ge N\Big(q - h(e_z + \mu_z(\delta)) + h(e_x + \mu_x(\delta))\Big) - 2\log\frac{2}{\delta^2}\,. \tag{72}$$

This, in turn, can be connected with the min-entropy bound on the one-shot quantum capacity that we recapitulated in Section 2, which reads

$$\forall\,\varepsilon > 0: \quad Q^\varepsilon(\Lambda) \ge \sup_{\eta\in(0,\sqrt{\varepsilon/2})}\left(H_{\min}^{\sqrt{\varepsilon/2}-\eta}(A|E)_\rho - 4\log\frac{1}{\eta} - 2\right)\,. \tag{73}$$

To connect inequalities Supplementary Equation 72 and Supplementary Equation 73, we make a variable transformation such that

$$3\delta + \delta' + 4\delta'' = \sqrt{\varepsilon/2} - \eta\,, \tag{74}$$

where

$$3\delta + \delta' + 4\delta'' = \delta\left(3 + \frac{1}{\sqrt{p_{\text{pass}}^z}} + \frac{4}{\sqrt{p_{\text{pass}}^x}}\right)\,. \tag{75}$$

Hence, we get

$$\forall\,\varepsilon > 0: \quad Q^\varepsilon(\Lambda) \ge \sup_{\eta\in(0,\sqrt{\varepsilon/2})} N\Big(q - h(e_z + \mu_z(\delta)) + h(e_x + \mu_x(\delta))\Big) - 2\log\frac{2}{\delta^2} - 4\log\frac{1}{\eta} - 2 \tag{76}$$

with

$$\delta = \frac{\sqrt{\varepsilon/2} - \eta}{3 + \frac{1}{\sqrt{p_{\text{pass}}^z}} + \frac{4}{\sqrt{p_{\text{pass}}^x}}} \tag{77}$$

and with μ_z and μ_x as in Supplementary Equation 67 and Supplementary Equation 69, respectively. This is the general version of our bound for the verification protocol.

To derive the form of the bound that we presented in the main article, we make two simplifications. Firstly, we consider the probability that both $\gamma \le e_x$ and $\lambda \le e_z$, and denothe this joint probability by p_{pass}. It bounds both probabilities from below, i.e. $p_{\text{pass}} \le p_{\text{pass}}^z$ and $p_{\text{pass}} \le p_{\text{pass}}^x$. We set $p = 1 - p_{\text{pass}}$. Thus, the bound Supplementary Equation 76 also holds with

$$\delta = \frac{\sqrt{\varepsilon/2} - \eta}{3 + \frac{5}{\sqrt{1-p}}}\,. \tag{78}$$

Secondly, we set $k = n = N$, and get

$$\mu_z(\delta) = \mu_x(\delta) = \mu(\delta) = \sqrt{\frac{2(N+1)}{N^2}\ln\frac{1}{\delta}}\,. \tag{79}$$

Inserting Supplementary Equation 78 and Supplementary Equation 79 into Supplementary Equation 76 gives us the form that we used in the main article.

Supplementary Note 6: Proof for the estimation protocol

Our estimation protocol has one essential difference to the verification protocol. In the verification protocol, the two error rates γ and λ that are measured do not enter the bound directly. Instead, they are compared with some maximally tolerated error rates e_x and e_z, and the bound is a function of these values. In the estimation protocol, there are no preset maximally tolerated error rates. Alice and Bob simply measure two error rates e_x and e_z, and the bound that they use is a function of these measured error rates. This may seem different to the verification protocol, but using a simple argument, we can see that the situation in the estimation protocol is analogous to the situation in the verification protocol. (This will also explain why we use the same notation as for the preset values e_x and e_z).

Suppose that Alice and Bob run the estimation protocol (see Protocol 1 of the main article) up to the point where they determine the error rates. For now, let us denote these error rates by γ and λ. Imagine that at that point, Alice and Bob decide that they actually wanted to make a test in which they check whether both $\gamma \le e_x$ and $\lambda \le e_z$ holds. However, in contrast to the verification protocol, where e_x and e_z are preset values, Alice and Bob say that they simply want to make the test for values of e_x and e_z that are exactly equal to the the error rates that they have just measured, $e_x = \gamma$ and $e_z = \lambda$. Obviously, if Alice and Bob design the test in this way, they will always pass the test. Moreover, the interpretation of the passing probability changes: it is no longer the probability that the measured error rates are below some preset values. Instead, it becomes the probability that in any run, the measured error rates stay below the rates that have been measured in this run (more precisely, it is a lower bound on it). This probability can be seen as a measure for the typicality of the protocol run, so we may denote it by p_{typical}. In Protocol 1 in the main article, we use the complementary probability $p = 1 - p_{\text{typical}}$. Using this argument, we can consider the state conditioned on passing a correlation test, just as in the case of the verification protocol.

For this reason, the same general form of the bound Supplementary Equation 76 with the same function δ as in Supplementary Equation 77 holds as for the verification protocol. However, in the estimation protocol, we use the measured error rates to infer the max-entropies $H_{\max}^{\varepsilon'}(Z|B)$ and $H_{\max}^{\varepsilon''}(X|B)$ for all qubits, rather than just on a part that has not been measured. Therefore, the functions μ_x and μ_z differ from the functions for the verification protocol. In order to derive the form of these functions, we again consider a slight generalization of the protocol that we considered in the main article. In the main article, we assumed that the N qubits that go through the channel are divided into $N/2$ qubits that are prepared and measured in the X-basis and $N/2$ qubits that are measured in the Z-basis. Here we assume that n qubits are measured in X and k qubits are measured in Z, with $n + k = N$. We denote the measured error rate in X by γ and the measured error rate in Z by λ. This is shown in Supplementary Figure 5. In order to bound $H_{\max}^{\varepsilon'}(Z|B)$ from λ, we follow the original derivation of Supplementary Equation 65 as in reference [15], adjusted to the situation shown in Supplementary Figure 5. For a detailed derivation, see also [8, 7].

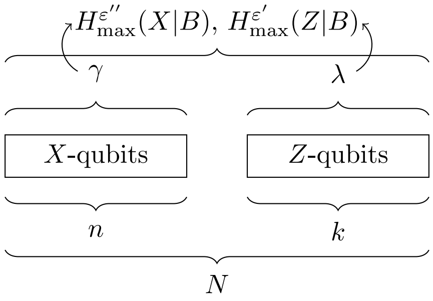

Supplementary Figure 5: Inference of the max-entropies in the estimation protocol. In the estimation protocol, the measured error rates are used to bound the max-entropies on the total system of all qubits. This is in contrast to the verification protocol, where the measured error rates were used to bound the max-entropies on only a part of the total system. This is why we cannot simply use the function μ as in Supplementary Equation 65 but need to derive them for this particular situation.

We consider the Gedankenexperiment in which all the bits have been measured in the Z-basis. We denote random variable of the error rate λ in Z in the Z-bits by $\Lambda = \Lambda_z$, the error rate in Z in the X-bits by Λ_x and the total error rate in Z by Λ_{tot}. Then it holds that

$$n\Lambda_x + k\Lambda_z = (n+k)\Lambda_{\text{tot}} \, . \tag{80}$$

The division of the qubits into X-qubits and Z-qubits is fully random. Therefore, the error number probabilities follow a hypergeometric distribution. This means that Serfling's bound [10] applies. Here, we use the particular

form presented in inequality (1.3) in [3].

$$\forall \nu > 0: \quad P[\sqrt{n}(\Lambda_x - \Lambda_{\text{tot}}) \geq \nu] \leq \exp\left(-2\nu^2 \frac{1}{1-\frac{n-1}{n+k}}\right) \tag{81}$$

Using Supplementary Equation 80, it is easy to show that

$$\sqrt{n}(\Lambda_x - \Lambda_{\text{tot}}) \geq \nu \iff \Lambda_{\text{tot}} \geq \Lambda_z + \frac{\sqrt{n}}{k}\nu \,. \tag{82}$$

Therefore, Supplementary Equation 81 is equivalent to

$$\forall \nu > 0: \quad P\left[\Lambda_{\text{tot}} \geq \Lambda_z + \frac{\sqrt{n}}{k}\nu\right] \leq \exp\left(-2\nu^2 \frac{1}{1-\frac{n-1}{n+k}}\right) \tag{83}$$

With the variable substitution

$$\nu = \frac{k}{\sqrt{n}}\mu_z \,, \quad \text{so that} \quad \mu_z = \frac{\sqrt{n}}{k}\nu \,, \tag{84}$$

we can write this as

$$\forall \mu_z > 0: \quad P\left[\Lambda_{\text{tot}} \geq \Lambda_z + \mu_z\right] \leq \exp\left(-2\left(\frac{k}{\sqrt{n}}\mu_z\right)^2 \frac{1}{1-\frac{n-1}{n+k}}\right) \tag{85}$$

$$= \exp\left(-2\frac{k^2(n+k)}{n(k+1)}\mu_z^2\right) . \tag{86}$$

According to Bayes' theorem, it holds that

$$P\left[\Lambda_{\text{tot}} \geq \Lambda_z + \mu_z | \Lambda_z \leq e_z\right] \leq \frac{P[\Lambda_{\text{tot}} \geq \Lambda_z + \mu_z]}{P[\Lambda_z \leq e_z]} \tag{87}$$

and thus

$$P\left[\Lambda_{\text{tot}} \geq \Lambda_z + \mu_z | \Lambda_z \leq e_z\right] \leq \frac{\varepsilon^2}{p^z_{\text{pass}}} \,, \tag{88}$$

where

$$\varepsilon = \exp\left(-\frac{k^2(n+k)}{n(k+1)}\mu_z^2\right) , \tag{89}$$

$$p^z_{\text{pass}} = P[\Lambda_z \leq e_z] \,. \tag{90}$$

In [15, 7], it was shown that inequality Supplementary Equation 88 implies that the state of the total system of $N = n + k$ qubits, conditioned on $\Lambda_z \leq e_z$, satisfies

$$H_{\max}^{\varepsilon/\sqrt{p^z_{\text{pass}}}}(Z|B)_\rho \leq Nh(e_z + \mu_z) \,, \tag{91}$$

where μ_z is solved for in Supplementary Equation 89,

$$\mu_z = \sqrt{\frac{n(k+1)}{k^2(n+k)} \ln\frac{1}{\varepsilon}} \,. \tag{92}$$

The derivation of μ_x is essentially analogous: The only difference is that n and k change their roles, and that p^x_{pass} replaces p^z_{pass}. Thus, we get

$$H_{\max}^{\varepsilon/\sqrt{p^x_{\text{pass}}}}(X|B)_\rho \leq Nh(e_x + \mu_x) \,, \tag{93}$$

where

$$\mu_x = \sqrt{\frac{k(n+1)}{n^2(n+k)} \ln\frac{1}{\varepsilon}} \,, \tag{94}$$

$$p^x_{\text{pass}} = P[\Gamma_x \leq e_x] \,. \tag{95}$$

To get the form that we use in the main article, we make again make two simplifications. As for the verification protocol, we bound p^x_{pass} and p^z_{pass} by a joint passing probability p_{typical} and set $p = 1 - p_{\text{typical}}$. Finally, we set $n = k = N/2$ and get

$$\mu_x = \mu_z = \mu = \sqrt{\frac{N+2}{N^2} \ln\left(\frac{3 + \frac{5}{\sqrt{1-p}}}{\sqrt{\varepsilon/2} - \eta}\right)} \,. \tag{96}$$

This completes the proof.

$\lvert\langle 0\lvert+\rangle\rvert^2$	0.495 ± 0.016
$\lvert\langle 0\lvert-\rangle\rvert^2$	0.505 ± 0.016
$\lvert\langle 1\lvert+\rangle\rvert^2$	0.505 ± 0.016
$\lvert\langle 1\lvert-\rangle\rvert^2$	0.495 ± 0.016
q	0.985 ± 0.047

Supplementary Table 1: Estimation of the preparation quality. The overlap between Z and X bases is estimated before the experiment. Subsequently, we use a very conservative value of $q = 0.9$ to account for a potential drift in our gates over time.

Supplementary Note 7: Experimental estimation of q

In order to estimate the preparation quality q using (8) of the main text the overlap of the Z and X bases needs to be evaluated. To this end the states $|+\rangle$ and $|-\rangle$ are prepared 1024 times using resonant microwaves pulses before being read out in the Z-basis. Note that this is equivalent to a preparation of $|0\rangle$ and $|1\rangle$ and a readout in the X-basis when done immediately (i.e., when not estimating the capacity of the idling operation). The single shot measurement outcomes are averaged together and the resulting voltages compared to calibration points corresponding to the preparation of $|0\rangle$ and $|1\rangle$ to determine the relevant populations. Results are shown in table 1, uncertainties correspond to the standard error of the mean for a binomial distribution with probability p equal to the measured overlap. Including noise in the measurement as an additional independent source of error leads to an insignificant increase in the uncertainty. It should be noted that all measured overlaps correspond to the expected 0.5 within the uncertainty. Using $\max_{i,j=0,1} |\langle j_Z | i_X \rangle|^2 = 0.505 \pm 0.016$, we find $q = 0.985 \pm 0.047$. In order to account for potential drift in our gates, we use a conservative value of $q = 0.9$ when calculating the capacity in the main text.

Supplementary References

[1] H. Barnum, E. Knill, and M. A. Nielsen. On quantum fidelities and channel capacities. *IEEE Trans. Inf. Theory*, 46(4):1317–1329, 2000.

[2] F. Buscemi and N. Datta. The quantum capacity of channels with arbitrarily correlated noise. *IEEE Trans. Inf. Theory*, 56(3):1447–1460, 2010.

[3] E. Greene and J. A. Wellner. Exponential bounds for the hypergeometric distribution. arXiv:1507.08298v2, 2015.

[4] R. König, R. Renner, and C. Schaffner. The operational meaning of min- and max-entropy. *IEEE Trans. Inf. Theory*, 55(9):4337–4347, 2009.

[5] C. Morgan and A. Winter. Pretty strong converse for the quantum capacity of degradable channels. *IEEE Trans. Inf. Theory*, 60(1):317–333, Jan 2014.

[6] M. A. Nielsen and I. L. Chuang. *Quantum Computation and Quantum Information.* Cambridge University Press, 2000.

[7] C. Pfister. *Decoherence estimation in Quantum Theory and Beyond.* PhD thesis, 2016.

[8] C. Pfister, N. Lütkenhaus, S. Wehner, and P. J. Coles. Sifting attacks in finite-size quantum key distribution. *New J. Phys.*, 18(5):053001, 2016.

[9] R. Renner. *Security of Quantum Key Distribution.* PhD thesis, ETH Zürich, 2005.

[10] R. J. Serfling. Probability inequalities for the sum in sampling without replacement. *Ann. Stat.*, 2(1):39–48, 1974.

[11] W. F. Stinespring. Positive functions on C*-algebras. *Proc. Amer. Math. Soc.*, 6(2), 1955.

[12] M. Tomamichel. *A Framework for Non-Asymptotic Quantum Information Theory.* PhD thesis, 2012.

[13] M. Tomamichel, M. Berta, and J. M. Renes. Quantum coding with finite resources. *Nat. Comm.*, 7(11419), 2016.

[14] M. Tomamichel, R. Colbeck, and R. Renner. Duality between smooth min- and max-entropies. *IEEE Trans. Inf. Theory*, 56(9):4674–4681, 2010.

[15] M. Tomamichel, C. Lim, N. Gisin, and R. Renner. Tight finite-key analysis for quantum cryptography. *Nat. Commun.*, 3(634), 2012.

[16] M. Tomamichel and R. Renner. Uncertainty relation for smooth entropies. *Phys. Rev. Lett.*, 106(11):110506, 2011.

[17] A. Vitanov, F. Dupuis, M. Tomamichel, and R. Renner. Chain rules for smooth min- and max-entropies. *IEEE Trans. Inf. Theory*, 59(5):2603–2612, 2013.

[18] M. Wilde. *Quantum Information Theory.* Cambridge University Press, 2013.
